# Detection of β-amyloid positivity in Alzheimer’s Disease Neuroimaging Initiative participants with demographics, cognition, MRI and plasma biomarkers

**DOI:** 10.1093/braincomms/fcab008

**Published:** 2021-02-02

**Authors:** Duygu Tosun, Dallas Veitch, Paul Aisen, Clifford R Jack, William J Jagust, Ronald C Petersen, Andrew J Saykin, James Bollinger, Vitaliy Ovod, Kwasi G Mawuenyega, Randall J Bateman, Leslie M Shaw, John Q Trojanowski, Kaj Blennow, Henrik Zetterberg, Michael W Weiner

**Affiliations:** 1 San Francisco Veterans Affairs Medical Center, San Francisco, CA, USA; 2 Department of Radiology and Biomedical Imaging, University of California San Francisco, San Francisco, CA, USA; 3 Alzheimer’s Therapeutic Research Institute (ATRI), Keck School of Medicine, University of Southern California, San Diego, CA, USA; 4 Department of Radiology, Mayo Clinic, Rochester, MN, USA; 5 School of Public Health and Helen Wills Neuroscience Institute, University of California, Berkeley, CA, USA; 6 Division of Epidemiology, Department of Health Sciences Research, Mayo Clinic, Rochester, MN, USA; 7 Department of Neurology, Mayo Clinic, Rochester, MN, USA; 8 Department of Radiology and Imaging Sciences, Center for Neuroimaging, Indiana University School of Medicine, Indianapolis, IN, USA; 9 Indiana Alzheimer Disease Center, Indiana University School of Medicine, Indianapolis, IN, USA; 10 Department of Medical and Molecular Genetics, Indiana University School of Medicine, Indianapolis, IN, USA; 11 Department of Neurology, Washington University School of Medicine, St. Louis, MO, USA; 12 Hope Center for Neurological Disorders, Washington University School of Medicine, St. Louis, MO, USA; 13 Knight Alzheimer’s Disease Research Center, Washington University School of Medicine, St. Louis, MO, USA; 14 Department of Pathology and Laboratory Medicine, Perelman School of Medicine, University of Pennsylvania, Philadelphia, PA, USA; 15 Department of Psychiatry and Neurochemistry, Institute of Neuroscience and Physiology, The Sahlgrenska Academy, University of Gothenburg, Gothenburg, Sweden; 16 Clinical Neurochemistry Laboratory, Sahlgrenska University Hospital, Mölndal, Sweden; 17 Department of Neurodegenerative Disease, UCL Institute of Neurology, London, UK; 18 UK Dementia Research Institute at UCL, London, UK

**Keywords:** Alzheimer’s, β-amyloid, MRI, PET, plasma

## Abstract

*In vivo* gold standard for the ante-mortem assessment of brain β-amyloid pathology is currently β-amyloid positron emission tomography or cerebrospinal fluid measures of β-amyloid_42_ or the β-amyloid_42_/β-amyloid_40_ ratio. The widespread acceptance of a biomarker classification scheme for the Alzheimer’s disease continuum has ignited interest in more affordable and accessible approaches to detect Alzheimer’s disease β-amyloid pathology, a process that often slows down the recruitment into, and adds to the cost of, clinical trials. Recently, there has been considerable excitement concerning the value of blood biomarkers. Leveraging multidisciplinary data from cognitively unimpaired participants and participants with mild cognitive impairment recruited by the multisite biomarker study of Alzheimer’s Disease Neuroimaging Initiative, here we assessed to what extent plasma β-amyloid_42_/β-amyloid_40_, neurofilament light and phosphorylated-tau at threonine-181 biomarkers detect the presence of β-amyloid pathology, and to what extent the addition of clinical information such as demographic data, *APOE* genotype, cognitive assessments and MRI can assist plasma biomarkers in detecting β-amyloid-positivity. Our results confirm plasma β-amyloid_42_/β-amyloid_40_ as a robust biomarker of brain β-amyloid-positivity (area under curve, 0.80–0.87). Plasma phosphorylated-tau at threonine-181 detected β-amyloid-positivity only in the cognitively impaired with a moderate area under curve of 0.67, whereas plasma neurofilament light did not detect β-amyloid-positivity in either group of participants. Clinical information as well as MRI-score independently detected positron emission tomography β-amyloid-positivity in both cognitively unimpaired and impaired (area under curve, 0.69–0.81). Clinical information, particularly *APOE* ε4 status, enhanced the performance of plasma biomarkers in the detection of positron emission tomography β-amyloid-positivity by 0.06–0.14 units of area under curve for cognitively unimpaired, and by 0.21–0.25 units for cognitively impaired; and further enhancement of these models with an MRI-score of β-amyloid-positivity yielded an additional improvement of 0.04–0.11 units of area under curve for cognitively unimpaired and 0.05–0.09 units for cognitively impaired. Taken together, these multi-disciplinary results suggest that when combined with clinical information, plasma phosphorylated-tau at threonine-181 and neurofilament light biomarkers, and an MRI-score could effectively identify β-amyloid+ cognitively unimpaired and impaired (area under curve, 0.80–0.90). Yet, when the MRI-score is considered in combination with clinical information, plasma phosphorylated-tau at threonine-181 and plasma neurofilament light have minimal added value for detecting β-amyloid-positivity. Our systematic comparison of β-amyloid-positivity detection models identified effective combinations of demographics, *APOE*, global cognition, MRI and plasma biomarkers. Promising minimally invasive and low-cost predictors such as plasma biomarkers of β-amyloid_42_/β-amyloid_40_ may be improved by age and *APOE* genotype.

## Teaser

Tosun *et al.* report a systematic comparison of β-amyloid positivity detection models, identifying effective combinations of demographics, *APOE* genotype, global cognitive measures, MRI and plasma biomarkers as promising minimally invasive and low-cost assessments to detect the β-amyloid positivity using florbetapir PET status as the ground-truth.

## Graphical Abstract

**Graphical Abstract:**
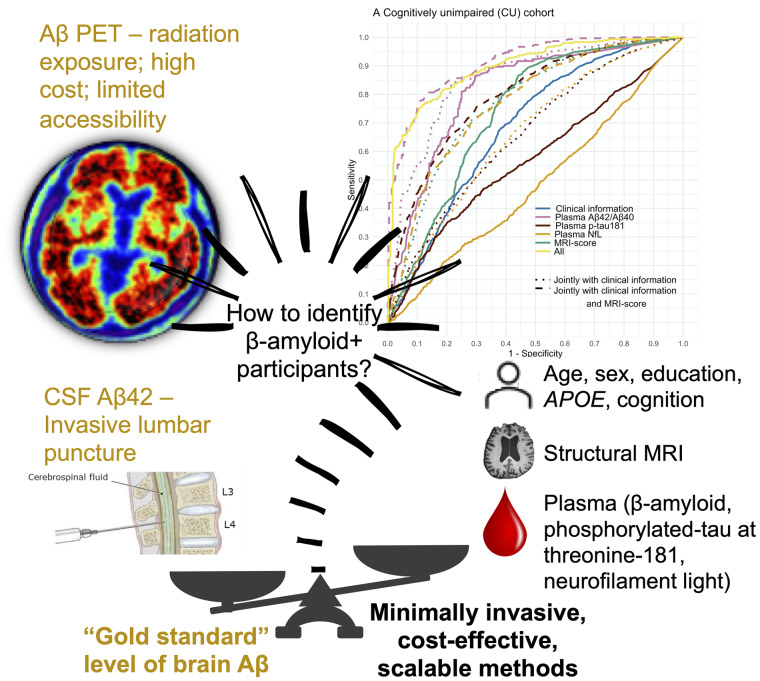

## Introduction

Alzheimer’s disease (AD), pathologically defined as the presence of plaques of β-amyloid (Aβ) protein, neurofibrillary tangles of tau protein and neurodegeneration (DeTure and Dickson, 2019), is the major cause of cognitive decline and dementia (2020). Currently, no treatment is approved that has been demonstrated to slow the progress of AD (Aisen, 2019). Historically, AD was diagnosed clinically through neurological and neuropsychological examinations to assess memory impairment and other thinking skills, judge functional abilities and identify behaviour changes, and exclude other causes than AD that could account for the dementia (McKhann *et al.*, 2011). The ‘gold-standard’ method to confirm the presence of AD pathology is pathological examination of brains at autopsy (DeTure and Dickson, 2019). Since the turn of the century, the ability to diagnose AD pathology in living people has been made possible by the development of radioligands for Aβ positron emission tomographic (PET) scans (Klunk *et al.*, 2004; Schilling *et al.*, 2016) and tau PET scans (Marquie *et al.*, 2015; Leuzy *et al.*, 2019), magnetic resonance imaging (MRI) for neurodegeneration (Frisoni *et al.*, 2010) and analysis of cerebrospinal fluid (CSF) for Aβ and tau species (Blennow, 2004; Holtzman, 2011). This has led to an *in vivo* biological framework of AD including Aβ, tau and neurodegeneration, based on the so-called A/T/N system (Jack *et al.*, 2018). Indeed, the descriptive A/T/N system places Aβ+ individuals firmly on the AD continuum, whereas individuals with Aβ- profiles are considered either normal or possessing non-AD pathologic changes (Jack *et al.*, 2018). Many trials, particularly the ones enrolling patients in earlier stages of disease, are therefore using either Aβ PET imaging or CSF Aβ_42_ levels as a critical step in clinical trial cohort enrichment (Sperling *et al.*, 2014; Honig *et al.*, 2018). Despite these advances, PET scans are quite expensive and not universally accessible. Although lumbar punctures are very safe (Peskind *et al.*, 2009), there continues to be reluctance to CSF sample collection in the patient and professional population (Moulder *et al.*, 2017). Therefore, there has been great interest in developing low cost, minimally invasive methods to detect AD Aβ pathology compared to PET scans and or CSF as the ‘gold standard’. Many publications (reviewed in Ashford *et al.*) have evaluated the role of demographics (Insel *et al.*, 2016; Tosun *et al.*, 2016; Jansen *et al.*, 2018; Buckley *et al.*, 2019; Ko *et al.*, 2019; Maserejian *et al.*, 2019), *APOE* ε4 (de Rojas *et al.*, 2018; Jansen *et al.*, 2018; Ten Kate *et al.*, 2018; Ba *et al.*, 2019; Buckley *et al.*, 2019), cognition (Mielke *et al.*, 2012; Burnham *et al.*, 2014; Kandel *et al.*, 2015; Burnham *et al.*, 2016; Insel *et al.*, 2016; Kim *et al.*, 2018; Lee *et al.*, 2018; Ba *et al.*, 2019; Brunet *et al.*, 2019; Maserejian *et al.*, 2019; Ansart *et al.*, 2020) and MRI measures (Tosun *et al.*, 2013, 2014, 2016; Ten Kate *et al.*, 2018; Petrone *et al.*, 2019; Ansart *et al.*, 2020; Ezzati *et al.*, 2020) to detect AD Aβ pathology. More recently, there has been considerable excitement concerning the value of assays of plasma Aβ species and related proteins (Burnham *et al.*, 2014, 2016; Kaneko *et al.*, 2014; Fandos *et al.*, 2017; Ovod *et al.*, 2017; Park *et al.*, 2017; de Rojas *et al.*, 2018; Nakamura *et al.*, 2018; Verberk *et al.*, 2018; Westwood *et al.*, 2018; Chatterjee *et al.*, 2019; Chen *et al.*, 2019; Goudey *et al.*, 2019; Lin *et al.*, 2019; Palmqvist *et al.*, 2019a,b; Park *et al.*, 2019; Perez-Grijalba *et al.*, 2019; Vergallo *et al.*, 2019), species of plasma tau, including phosphorylated tau (p-tau) forms (Mielke *et al.*, 2018; Palmqvist *et al.*, 2019b; Barthélemy *et al.*, 2020; Janelidze *et al.*, 2020a; Karikari *et al.*, 2020; Palmqvist *et al.*, 2020; Thijssen *et al.*, 2020) and plasma neurofilament light (NfL) (Palmqvist *et al.*, 2019b; Thijssen *et al.*, 2020) to detect AD Aβ pathology. The first reports of reproducible high precision, high accuracy tests of plasma Aβ_42_/Aβ_40_ indicated high sensitivity and specificity for Aβ plaques as measured by mass spectrometry (Ovod *et al.*, 2017; Nakamura *et al.*, 2018). Subsequently, plasma measures of p-tau at residues 181 (Mielke *et al.*, 2018) and 217 (Barthélemy *et al.*, 2020; Palmqvist *et al.*, 2020) indicated good performance relative to Aβ plaques and tau tangles. The performance of these tests is being evaluated and has been shown to detect PET Aβ-positivity conversion (Schindler *et al.*, 2019), to be associated with cognitive decline and to correlate with AD pathology (Janelidze *et al.*, 2020a). If proven useful, these alternative approaches to detect AD Aβ pathology may play an important role in drug discovery and in accelerating identification of risk factors for AD with greater precision.

For optimal and generalizable operationalization of such imputation approaches for the presence of AD Aβ pathology, it is important to assess the independent and added value of each class of predictors (e.g. demographics, *APOE* ε4, cognition, plasma biomarkers, MRI, etc.) and the differences in their classification performances at different clinical stages. The Alzheimer’s Disease Neuroimaging Initiate (ADNI) is a large, multi-site, longitudinal study aimed at validating biomarkers for AD clinical trials (Weiner *et al.*, 2017). ADNI participants have Aβ PET scans, lumbar punctures for CSF and blood drawn for plasma studies, therefore allowing for a head-to-head comparison. This study specifically aimed to assess (i) to what extent plasma Aβ_42_/Aβ_40_, NfL and p-tau181 biomarkers detect the presence of AD Aβ pathology (i.e. Aβ-positivity); (ii) to what extent the addition of demographic data, *APOE* genotype and cognitive assessments and (iii) MRI can assist plasma biomarkers in detecting Aβ-positivity and (iv) to what extent the stage of clinical diagnosis affects these relationships.

## Materials and methods

### Study design

Data used in the preparation of this article were obtained from the ADNI database (adni.loni.usc.edu). The ADNI was launched in 2003 as a public–private partnership, led by Principal Investigator Michael W. Weiner, MD. The primary goal of ADNI has been to test whether serial MRI, PET, other biological markers and clinical and neuropsychological assessment can be combined to measure the progression of mild cognitive impairment (MCI) and early AD. Up-to-date information are available at www.adni-info.org.

### Cohort

Subjects of this study were ADNI participants with known PET Aβ status and with plasma biomarker assessments for p-tau181, and NfL, clinical assessments and structural MRI within 6 months of Aβ PET imaging. A subset of the main study cohort also had plasma biomarker assessment for Aβ_42_/Aβ_40_. The primary focus of this study was to assess imputation of Aβ positivity from single time-point observations of clinical, neuroimaging and plasma biomarker data; therefore, a cross-sectional study design was used. Although longitudinal biomarkers, neuroimaging and clinical data are available for many ADNI participants, we considered only data from the first time-point with complete clinical, neuroimaging and biomarker assessments for each participant to avoid circular model training and assessment. Clinical assessment closest in time to Aβ PET imaging was used to define cognitively unimpaired (CU) and cognitively impaired (CI) diagnostic groups. The diagnostic criteria for ADNI participants were described previously (Petersen *et al.*, 2010). Participant selection was made *a priori* from all ADNI subjects based on the availability of complete cross-sectional data as of 30 June 2020.

### PET Aβ status

Mean tracer uptake in the cerebellar grey and white matter was computed and used as reference to generate whole-brain standardized uptake value ratio (SUVR) maps of florbetapir PET scans (Jagust *et al.*, 2015). A composite region-of-interest consisting of middle frontal, anterior cingulate, posterior cingulate, inferior parietal, precuneus, supramarginal, middle temporal and superior temporal regions was used to compute a global SUVR for florbetapir. A threshold of SUVR ≥1.11 for florbetapir (Landau *et al.*, 2013) was then used to determine the status of PET Aβ.

### Demographics data

Age at florbetapir PET imaging, sex and years of education were included as demographic characteristics of each participant.

### Apolipoprotein E genotyping

*APOE* genotyping was done by the ADNI Genetics Core using DNA from blood samples as detailed in Supplementary Material. *APOE* ε4 carrier status was considered as a predictor of Aβ-positivity in this study.

### Global cognitive assessments

ADNI participants were assessed with a wide spectrum of clinical and cognitive tests (Weiner *et al.*, 2017). In this study, we limited the global cognitive assessments to the Clinical Dementia Rating—Sum of Boxes (CDR–SB), the Alzheimer’s Disease Assessment Scale—Cognitive subscale 13-item (ADAS–Cog) and the Mini–Mental State Examination (MMSE) based on a 30-point questionnaire.

### Plasma sample collection

Blood samples were obtained by venipuncture in EDTA tubes for plasma by following the ADNI protocol (Kang *et al.*, 2015). Within 60 min, the samples were centrifuged at 3000 r.p.m. at room temperature, aliquoted and stored at −80°C. Samples underwent two freeze/thaws. Further details are provided in Supplementary Material.

### Plasma Aβ_42_ and Aβ_40_

Plasma Aβ isoform concentrations were determined using immunoprecipitation combined with liquid chromatography tandem mass spectrometry (LC-MS/MS) as described previously (Ovod *et al.*, 2017). Plasma aliquots were thawed at 21°C/800 RPM for 10 min and centrifuged at 21°C/10 000 RCF for 5 min prior to immunoprecipitation. Targeted Aβ_42_ and Aβ_40_ isoforms were immunoprecipitated with an anti-Aβ mid-domain antibody (HJ5.1) using a KingFisher (Thermo) automated immunoprecipitation platform. Immuno-enriched fractions were subsequently digested with Lys-N protease, generating Aβ_28-42_ and Aβ_28-40_ species, which were measured by LC-MS/MS (Ovod *et al.*, 2017). Absolute Aβ isoform concentrations were determined with a 15 N-labelled internal standard for each isoform. The total levels of Aβ_42_ and Aβ_40_ were used to calculate the Aβ_42_/Aβ_40_ ratio.

### Plasma p-tau181

Plasma p-tau181 was analysed by the Single-molecule array (Simoa) technique (Quanterix, Billerica, MA), using an assay developed in the Clinical Neurochemistry Laboratory, University of Gothenburg, Sweden (Karikari *et al.*, 2020). The assay uses a combination of two monoclonal antibodies (Tau12 and AT270) and measures N-terminal to mid-domain forms of p-tau181 (Karikari *et al.*, 2020). Calibrators were run as duplicates, whereas plasma samples were measured in singlicate. All the available samples were analysed in a single batch.

### Plasma NfL

Plasma NfL was analysed by the Simoa technique (Quanterix, Billerica, MA). The assay uses a combination of monoclonal antibodies, and purified bovine NfL as a calibrator. Calibrators were run as triplicates, whereas plasma samples were measured in singlicate. All the available samples were analysed in a single batch.

### MRI-score for Aβ-positivity

3T MRI data included a 3D MP-RAGE or IR-SPGR T1-weighted acquisition, as described online (http://adni.loni.usc.edu/methods/documents/mri-protocols). We employed a previously proposed methodology for assessing brain Aβ positivity status (Lang *et al.*, 2019). Briefly, 972 ADNI patients with structural MRI scans and with known Aβ status based on either CSF or Aβ PET imaging were used to train a deep learning model. The deep learning model training cohort included individuals at different clinical stages (CU, subjective memory complaint, early/late MCI and dementia), but excluding the participants of this study with plasma biomarker data. The method yields a probabilistic score of Aβ-positivity between 0 and 1.

### Statistical analysis

All analyses were performed on CU and CI data separately.

Demographic, clinical and biomarker characteristic differences between Aβ+ and Aβ− participants were described using two-sample *t*-test and the *χ*^2^ test for continuous and categorical variables, respectively.

Demographic characteristics (age, sex and years of education), *APOE* genotype, cognitive scores (MMSE, ADAS–Cog, and CDR–SB), plasma Aβ_42_/Aβ_40_, p-tau181 and NfL levels, and derived MRI-score were used as inputs to construct random forest (RF) classifiers to detect the Aβ-positivity using florbetapir PET status as the ground-truth. RF approach was pre-selected based on the classification performances that are previously reported (Fernández-Delgado et al., 2014) and flexibility of RF models to a mixture of numerical (age, years of education, cognitive scores, plasma levels and MRI-score) and categorical (sex and *APOE* genotype) features. A reference RF classifier was constructed from demographics and cognitive scores, referred as the clinical information here on. A second reference RF classifier was also constructed from MRI-score alone. To assess the added value of each class of variables (i.e. clinical, plasma and MRI classes), additional RF classifiers were constructed from (i) each plasma marker alone, (ii) each plasma marker jointly with clinical features, (iii) MRI-score jointly with clinical features and (iv) each plasma marker jointly with clinical features and MRI-score.

The RF model construction was repeated 10 times using different random seeds, and the average model performance was reported. Each data set (CU and CI data sets) was randomly divided into training and test data sets, using non-overlapping 80%/20% split. Each data set used the same partitioning for all classifiers for an unbiased comparison between classifiers (Vanschoren *et al.*, 2012). The models were built on each training split, and the performance on the test data sets was evaluated, and this process was repeated 10 times. Performance was presented as mean and standard deviation over the model runs. We generated sensitivity–specificity curves based on model classifications on the test data. For each sensitivity–specificity curve, we also computed the area under curve (AUC) values. A confidence interval of 95% was chosen. AUC of two classifiers was compared with DeLong test (DeLong *et al.*, 1988). Additionally, we computed accuracy, sensitivity, specificity, positive predictive value (PPV) and negative predictive value (NPV) on each set of model classifications at classifier probability cut-off of 0.5.

Finally, for RF models with multiple variables, the mean decrease in accuracy caused by a variable was determined based on the out-of-bag error estimates. The more the accuracy of the RF decreases due to the exclusion of a single variable, the more important that variable was deemed for the classification of the data.

The main analyses reported below with PET Aβ-positivity as the gold-standard for Aβ-positivity were repeated with CSF Aβ-positivity and the results are shown in Supplementary Fig. 1. Results from another secondary analysis are also shown in Supplementary Fig. 2, in which each classifier model was considered in a sub-sample constraint by the plasma Aβ_42_/Aβ_40_ cohort where all relevant data was available, yielding a fixed sample size across all classifier models. Finally, the main analyses were repeated by restricting clinical information to age and *APOE* genotype (Supplementary Fig. 3).

All analyses were performed using the R language and environment for statistical computing version 4.0.1 (R Foundation for Statistical Computing).

## Data availability

Data used in this study has been made publicly available by the ADNI in the Laboratory of Neuro Imaging (LONI) database.

## Results

The results of plasma Aβ_42_/Aβ_40_ for nine CU and nine CI participants failed quality control at measurement. No outliers (i.e. > 4 standard deviations of the mean) were detected in the plasma Aβ_42_/Aβ_40_ measurements. Samples from three CU and one CI participants were measured below the lower limit of quantification of 1.0 pg/ml for plasma p-tau181. We identified additional five CU and five CI participants with outlier values of plasma p-tau181 levels, who were discarded from subsequent analyses. Analytical sensitivity for plasma NfL was <1.0 pg/ml, and no sample contained NfL levels in plasma below the limit of detection, but 5 CUs and 11 CIs were excluded from our analyses due to outlier plasma NfL values. Participants with dementia were excluded for two main reasons. First, 91% of the AD participants (*n *=* *235) with plasma NfL and plasma p-tau181 biomarker data were PET Aβ-positive. An unbiased classification performance analysis with a prevalence of 91% Aβ-positivity would have required a sample size of >500 (Hanczar *et al.*, 2010). Second, cross-sectional plasma Aβ_42_/Aβ_40_ data was available only for non-demented participants. The final main study cohort was composed of 333 CU and 519 CI elderly individuals. Participant characteristics are reported in Table 1.

**Table 1:** Sample demographics per clinical diagnostic group

	CU Aβ−	CU Aβ+	*P* value	CI Aβ−	CI Aβ+	*P* value
*Main cohort*						
*N*	224	109		230	289	
Age (years)	72.8 ± 6.2	74.6 ± 5.3	*0.01*	70.3 ± 7.9	73.3 ± 6.8	*<10^−5^*
Sex (Female %)	52%	36%	*0.005*	56%	56%	
Education (years)	16.8 ± 2.5	15.9 ± 2.8	*0.003*	16.3 ± 2.5	15.9 ± 2.9	*0.024*
*APOE* ε4 (carrier %)	21%	43%	*<10^−4^*	23%	66%	*<10^−15^*
MMSE	29.1 ± 1.3	28.9 ± 1.1		28.4 ± 1.6	27.6 ± 1.8	*<10^−6^*
CDR-SB	0.06 ± 0.2	0.1 ±0.3	*0.03*	1.3 ± 0.8	1.6 ± 0.9	*<10^−4^*
ADAS-Cog	5.5 ± 3.1	6.3 ± 3.0	*0.02*	7.8 ± 3.8	10.4 ± 4.6	*<10^−10^*
Plasma NfL (pg/ml)	35.4 ± 15.8	39.4 ± 15.8	*0.03*	35.0 ± 18.7	43.3 ± 19.8	*<10^−5^*
Plasma p-tau181 (pg/ml)	14.7 ± 10.6	16.9 ± 7.8		13.6 ± 8.6	21.6± 10.7	*<10^−14^*
*Plasma Aβ_42_/Aβ_40_ sub-cohort*						
*N*	50	37		40	46	
Age (years)	71.9 ± 6.1	75.3 ± 5.2	*0.009*	70.0 ± 7.9	73.1 ± 6.9	
Sex (Female %)	50%	33%		52%	51%	
Education (years)	16.8 ± 2.6	16.1 ± 2.4		16.4 ± 2.5	16.0 ± 3.0	
*APOE* ε4 (carrier %)	14%	51%	*0.001*	22%	63%	*0.002*
MMSE	29.2 ± 1.0	28.9 ± 1.0		28.5 ± 1.3	27.6 ± 2.0	*0.04*
CDR-SB	0.04 ± 0.1	0.11 ± 0.2		0.8 ± 0.2	0.7 ± 0.2	
ADAS-Cog	5.5 ± 2.7	6.5 ± 3.1		7.0 ± 3.0	8.4 ± 3.4	
Plasma NfL (pg/ml)	32.1 ± 15.8	36.1 ± 12.3		30.8 ± 11.3	37.7 ± 14.7	*0.04*
Plasma p-tau181 (pg/ml)	13.5 ± 10.1	18.8 ± 7.7	*0.01*	14.5 ± 10.0	18.7 ± 7.6	
Plasma Aβ_42_/Aβ_40_	0.12 ± 0.01	0.11 ± 0.01	*<10^-6^*	0.13 ± 0.01	0.11 ± 0.009	*<10^−10^*

CU, cognitively unimpaired elderly; CI, elderly individuals with mild cognitive impairment; *APOE*, apolipoprotein E; MMSE, Mini-Mental State Examination; CDR-SB, Clinical Dementia Rating—Sum of Boxes; ADAS-Cog, Alzheimer’s Disease Assessment Scale—Cognitive subscale 13-item; NfL, neurofilament light.

**Figure 1:**
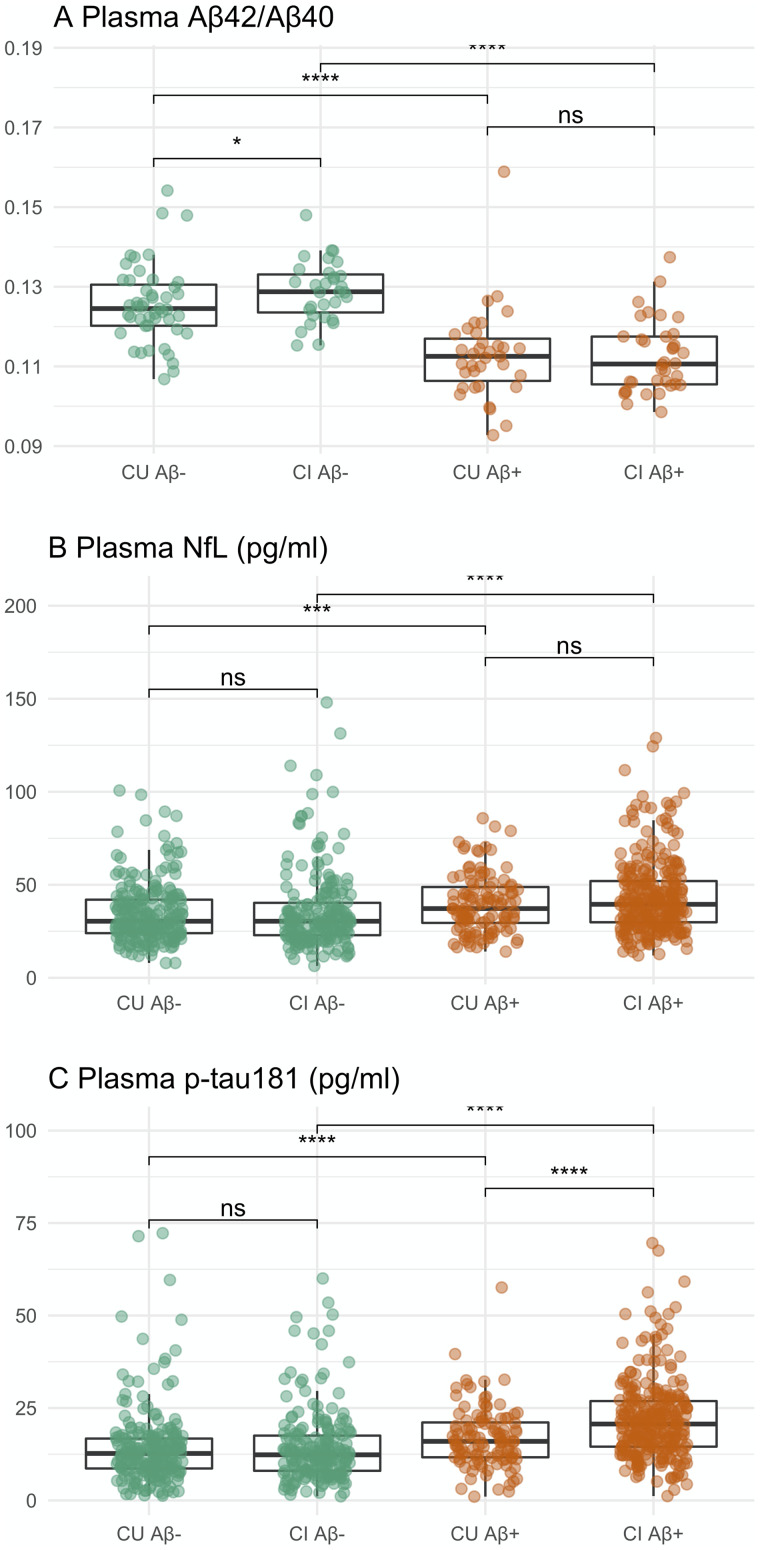
**Plasma (A) Aβ_42_/Aβ_40_, (B) NfL concentrations and (C) p-tau181 concentrations categorized by clinical diagnosis and CSF Aβ-positivity.** Plasma Aβ_42_/Aβ_40_ data was available for 173 individuals (Aβ− CU, *n *=* *50; Aβ+ CU, *n *=* *37; Aβ− CI, *n *=* *40; Aβ+ CI, *n *=* *46). Plasma p-tau181 and NfL data included 852 individuals (Aβ− CU, *n *=* *224; Aβ+ CU, *n *=* *109; Aβ− CI, *n *=* *230; Aβ+ CI, *n *=* *289). Unpaired two-samples *t*-test uncorrected significance levels at ****P < 0.00001; ***P < 0.0001; **P < 0.001; ns: *P* ≥ 0.5. CU, cognitively unimpaired elderly; CI, elderly individuals with mild cognitive impairment.

In brief, 33% of CU participants in the main study cohort were PET Aβ+. The frequency of *APOE* ε4 allele was higher among Aβ+ CUs compared to Aβ− CUs. Compared to Aβ− CUs, Aβ+ CUs were older with fewer females and had significantly fewer years of education, greater CDR-SB and ADAS-Cog scores, as well as greater plasma NfL levels (Fig. 1). Plasma p-tau181 levels were marginally higher in Aβ+ CUs compared to Aβ− CUs (*P* = 0.057). When controlled for age differences, Aβ− CUs and Aβ+ CUs did not differ in ADAS-Cog scores and plasma NfL levels. Demographic and clinical characteristics of CUs in the plasma Aβ_42_/Aβ_40_ sub-cohort did not differ from those of the main study CUs. Within the plasma Aβ_42_/Aβ_40_ sub-cohort, Aβ+ CUs had lower plasma Aβ_42_/Aβ_40_ compared to Aβ− CUs (Fig. 1; *P* *<* *10^−6^*).

In total, 57% of CI participants in the main study cohort were PET Aβ+. Aβ+ CIs were older than Aβ− CIs with fewer years of education and a higher frequency of *APOE* ε4 allele. Compared to Aβ− CIs, Aβ+ CIs had greater clinical symptoms, with lower MMSE and higher CDR-SB and ADAS-Cog scores. Aβ+ CIs had significantly higher plasma p-tau181 and plasma NfL levels than Aβ− CIs (Fig. 1). Aβ− versus Aβ+ CI group differences in clinical scores and plasma levels were significant after controlling for age differences. Compared to the CIs in the main study cohort, CIs in the plasma Aβ_42_/Aβ_40_ sub-cohort had lower symptom severity (i.e. mean CDR-SB of 1.4 versus 0.7 with *P < 10*^−^^*15*^ and mean ADAS-Cog of 9.2 versus 7.8 with *P *=* *0.002) and lower plasma NfL levels (39.5 versus 34.5 pg/ml with *P *=* *0.01). Within the plasma Aβ_42_/Aβ_40_ sub-cohort, Aβ+ CIs had significantly lower plasma Aβ_42_/Aβ_40_ compared to Aβ− Cis (Fig. 1; *P < 10*^−^^*10*^).

**Figure 2:**
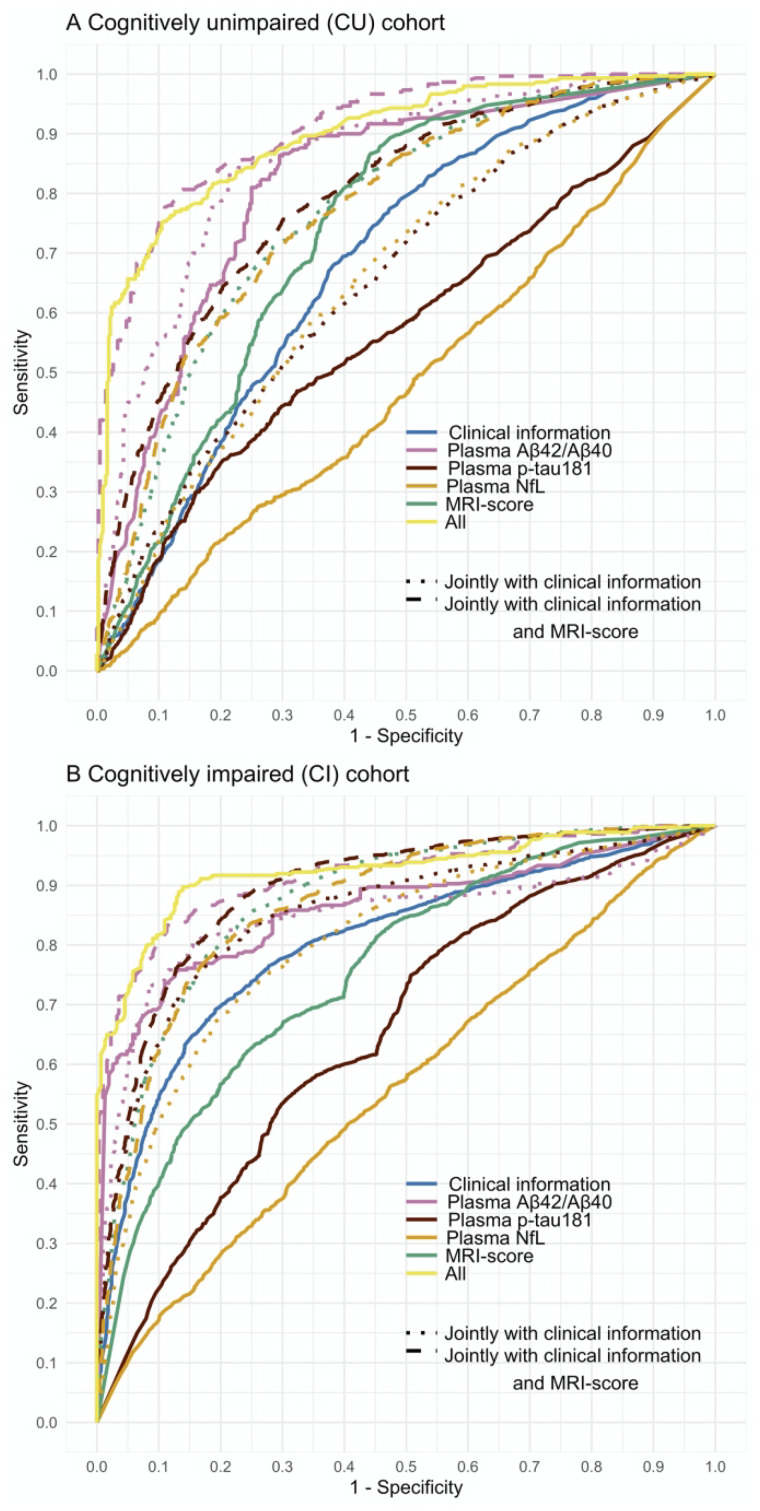
**Receiver-operating characteristic (ROC) analysis of Aβ positivity prediction in an ADNI cohort of (A) cognitively unimpaired (CU) and (B) cognitively impaired (CI) elderly individuals.** Optimized ROC curves for classifiers constructed separately and jointly with demographic information (age, sex and years of education), *APOE*, clinical scores, plasma biomarkers (Aβ_42_/Aβ_40_, p-tau181 and NfL), and structural MRI-score when predicting Aβ-positivity using florbetapir PET as the ground truth in the ADNI study (*n* = 333 CUs and *n* = 519 CIs). To assess the added value of each class of variables (i.e. clinical, plasma and MRI classes), additional RF classifiers were constructed from (i) each plasma marker alone, (ii) each plasma marker jointly with clinical features, (iii) MRI-score jointly with clinical features and (iv) each plasma marker jointly with clinical features and MRI-score. Models including plasma Aβ_42_/Aβ_40_ were tested and validated in a cohort of *n* = 87 CUs and *n* = 86 CIs due to limited availability of plasma Aβ_42_/Aβ_40_ data. Error bars indicate union of 95% CIs from cross-validation iterations.

**Figure 3:**
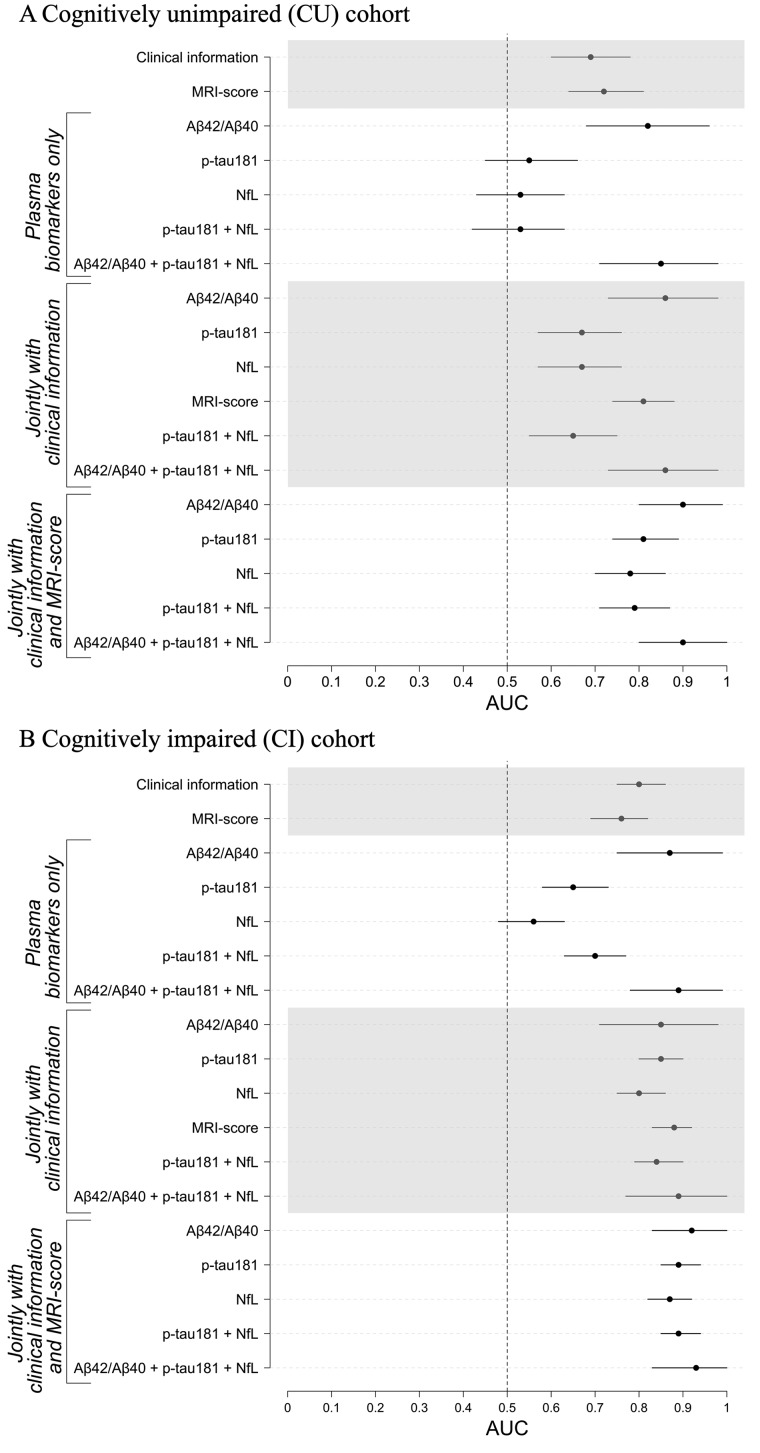
**Classifier performance metrics of Aβ positivity prediction in (A) cognitively unimpaired (CU) individuals and B) individuals with mild cognitive impairment (CI).** Area under the curve (AUC) estimates with ±2 × standard variation error bars from cross-validation iterations are shown for classifiers constructed separately and jointly with demographic information (age, sex and years of education), *APOE*, clinical scores, plasma biomarkers (Aβ_42_/Aβ_40_, p-tau181 and NfL) and structural MRI-score when predicting Aβ-positivity using florbetapir PET as the ground truth in the ADNI study (*n* = 333 CUs and *n* = 519 CIs). To assess the added value of each class of variables (i.e. clinical, plasma and MRI classes), additional RF classifiers were constructed from (i) each plasma marker alone, (ii) each plasma marker jointly with clinical features, (iii) MRI-score jointly with clinical features and (iv) each plasma marker jointly with clinical features and MRI-score. Models including plasma Aβ_42_/Aβ_40_ were tested and validated in a cohort of *n* = 87 CUs and *n* = 86 CIs due to limited available of plasma Aβ_42_/Aβ_40_ data. Error bars indicate union of 95% CIs from cross-validation iterations.

Performance of classifiers differentiating Aβ+ and Aβ− CU participants is shown and summarized in Figs. 2a and 3a and Table 2. A classifier constructed with only clinical information (i.e. demographics, *APOE* ε4 carrier status and global cognitive assessments) and a classifier constructed with only the MRI-score had similar performances (i.e. DeLong *P* = 0.06) with an accuracy of 67–68% in differentiating Aβ+ CUs and Aβ− CUs (Supplementary Fig. 4). Of these two classifiers, the MRI-score yielded better AUC (0.74 versus 0.69) reflected in higher NPV of MRI-score (76% versus 68%) and poor sensitivity of clinical information (3% versus 46%). When considered alone and together, plasma p-tau181 and plasma NfL did not differentiate Aβ+ and Aβ− CUs better than chance (Table 2; column (A)). In contrast, plasma Aβ_42_/Aβ_40_ alone differentiated Aβ+ CUs from Aβ− CUs with an accuracy of 72%, a PPV of 69% and an NPV of 76%, yielding an AUC of 0.80. The overall performance of plasma Aβ_42_/Aβ_40_ only classifier was similar to the performance of a classifier using MRI score and clinical information jointly (i.e. AUC of 0.80; DeLong *P *=* *0.53), with plasma Aβ_42_/Aβ_40_ having slightly better PPV (69% versus 65%), whereas the multi-disciplinary MRI score and clinical information jointly having slightly better accuracy (i.e. 75% versus 72%) and NPV (i.e. 78% versus 76%). All three plasma biomarkers jointly differentiated Aβ+ CU and Aβ− CU at an improved accuracy of 77%, a PPV of 77% and an NPV of 80%, yielding an AUC of 0.83, but this was not significantly different than the performance of plasma Aβ_42_/Aβ_40_ alone classification (DeLong *P* = 0.09).

**Table 2:** Performance of classifier models in classifying Aβ+ CU individuals

	(A) Plasma biomarkers						(B) Clinical information with and without plasma biomarkers						(C) MRI—score with and without clinical information and plasma biomarkers					
	AUC^a^	Acc	PPV	NPV	Sens	Spec	AUC^a^	Acc	PPV	NPV	Sens	Spec	AUC^a^	Acc	PPV	NPV	Sens	Spec
MRI score													0.74 [0.66, 0.82]	0.67 ± 0.04	0.48 ± 0.06	0.76 ± 0.03	0.46 ± 0.11	0.77 ± 0.04
Clinical information^b^							0.69 [0.60, 0.78]	0.68 ± 0.01	0.45 ± 0.28	0.68 ± 0.01	0.03 ± 0.03	0.98 ± 0.01	0.80 [0.72, 0.87]	0.75 ± 0.02	0.65 ± 0.06	0.78 ± 0.02	0.48 ± 0.09	0.88 ± 0.05
Aβ_42_/Aβ_40_	0.80 [0.65, 0.94]	0.72 ± 0.07	0.69 ± 0.12	0.76 ± 0.08	0.64 ± 0.18	0.77 ± 0.13	0.86 [0.73, 0.98]	0.79 ± 0.05	0.77 ± 0.08	0.81 ± 0.06	0.71 ± 0.11	0.84 ± 0.07	0.90 [0.80, 1.00]	0.83 ± 0.04	0.84 ± 0.08	0.83 ± 0.05	0.74 ± 0.10	0.89 ± 0.06
p-tau181	0.55^c^ [0.45, 0.66]	0.62 ± 0.02	0.39 ± 0.04	0.71 ± 0.02	0.37 ± 0.07	0.73 ± 0.05	0.69 [0.60, 0.78]	0.69 ± 0.01	0.58 ± 0.29	0.69 ± 0.01	0.07 ± 0.06	0.98 ± 0.03	0.80 [0.73, 0.88]	0.76 ± 0.03	0.69 ± 0.07	0.78 ± 0.02	0.46 ± 0.06	0.90 ± 0.04
NfL	0.54^c^ [0.44, 0.64]	0.57 ± 0.03	0.31 ± 0.05	0.68 ± 0.02	0.31 ± 0.08	0.69 ± 0.04	0.68 [0.59, 0.77]	0.68 ± 0.01	0.51 ± 0.33	0.68 ± 0.01	0.03 ± 0.02	0.98 ± 0.02	0.79 [0.71, 0.87]	0.74 ± 0.03	0.64 ± 0.06	0.78 ± 0.02	0.45 ± 0.06	0.88 ± 0.04
p-tau181 + NfL	0.53^c^ [0.42, 0.63]	0.60 ± 0.04	0.34 ± 0.07	0.69 ± 0.02	0.26 ± 0.08	0.76 ± 0.05	0.65 [0.56, 0.75]	0.69 ± 0.03	0.53 ± 0.10	0.72 ± 0.02	0.25 ± 0.08	0.90 ± 0.04	0.80 [0.72, 0.88]	0.76 ± 0.03	0.66 ± 0.08	0.80 ± 0.02	0.54 ± 0.08	0.87 ± 0.05
Aβ_42_/Aβ_40_ + p-tau181 + NfL	0.83 [0.68, 0.97]	0.77 ± 0.06	0.77 ± 0.13	0.80 ± 0.06	0.70 ± 0.12	0.83 ± 0.14	0.85 [0.72, 0.98]	0.81 ± 0.04	0.82 ± 0.08	0.83 ± 0.06	0.73 ± 0.11	0.87 ± 0.08	0.91 [0.81, 0.99]	0.82 ± 0.06	0.90 ± 0.08	0.79 ± 0.07	0.63 ± 0.14	0.95 ± 0.04

To assess the added value of each class of variables (i.e. clinical, plasma and MRI classes), additional RF classifiers were constructed from (i) each plasma marker alone, (ii) each plasma marker jointly with clinical features, (iii) MRI-score jointly with clinical features and (iv) each plasma marker jointly with clinical features and MRI-score.

a 95% Confidence intervals.

b Demographics: Age, sex, years of education and *APOE* ε4 status; Global cognitive assessments: MMSE, ADAS-Cog and CDR–SB.

c The confidence interval includes the axis *y* = *x*, suggesting that the classifier was not better than chance.

When combined with clinical information (Table 2; column (B)), the predictive performance of the plasma p-tau181 and plasma NfL improved but not beyond the performance of the classifier constructed from clinical information alone (i.e. DeLong *P* = 0.18 and *P* = 0.08, respectively). Adding clinical information to the plasma Aβ_42_/Aβ_40_ classifier yielded better differentiation of Aβ+ CU and Aβ− CU cases with an accuracy of 79%, PPV of 77%, NPV of 81% and an AUC 0.86, with the greatest improvement in the PPV compared to plasma Aβ_42_/Aβ_40_ only and clinical information only classifiers. Further enhancing plasma NfL and plasma p-tau181 with the MRI score in addition to the clinical information improved classification accuracy by 5–8%, PPV by 13–22%, NPV by 8–11% and AUC by 0.10–0.14 (DeLong *P* < 10^−14^ and *P* < 10^−21^, respectively) but this was not better than the classifier constructed with the MRI-score and clinical information (i.e. DeLong *P* = 0.08 and *P* = 0.46, respectively) or the classifier based on plasma Aβ_42_/Aβ_40_ only (i.e. DeLong *P* = 0.07 and *P* = 0.88, respectively) as reported in Table 2 (column (C)). Of the three plasma biomarkers, Aβ_42_/Aβ_40_ in combination with the MRI-score and clinical information performed the best with an accuracy of 83% and AUC of 0.90, with a well-balanced PPV of 84% and NPV of 83%, which was significantly better than the performance of Aβ_42_/Aβ_40_ alone (i.e. DeLong *P* < 10^−4^) or in combination with clinical information (i.e. DeLong *P* = 0.02).

The full classifier model including all three plasma biomarkers, the MRI-score and clinical information had an accuracy of 82%, with a PPV of 90% and NPV of 79%. However, this was not significantly different from the classifier model with plasma Aβ_42_/Aβ_40_, MRI-score and clinical information (DeLong *P* = 0.61), suggesting minimal added value of plasma NfL and plasma p-tau181. The most significant variables in a decreasing order of importance based on mean decrease in accuracy analysis were plasma Aβ_42_/Aβ_40_, MRI-score, *APOE* ε4 status, MMSE, years of education and sex.

Performance of classifiers differentiating Aβ+ and Aβ− CI participants is shown and summarized in Figs. 2b and 3b and Table 3. Both clinical information-based and MRI-score-based classifiers were performed moderately well in differentiating Aβ+ and Aβ− CIs with an AUC of 0.81 and 0.76, accuracy of 74% and 67%, PPV of 76% and 70% and NPV of 73% and 63% (Supplementary Fig. 4), respectively. The MRI-score together with clinical information achieved an AUC of 0.88, with an accuracy of 81%, PPV of 82% and NPV of 80%, performing significantly better than clinical information only (DeLong *P* < 10^−15^) or MRI-score only (DeLong *P* < 10^−39^) models. In contrast to CU data, both plasma Aβ_42_/Aβ_40_ and plasma p-tau181, but not plasma NfL, separately detected Aβ-positivity in CIs with an average accuracy of 77% and 58%, PPV of 79% and 63%, NPV of 76% and 52%, yielding AUCs of 0.87 and 0.64, respectively. Enhancement with clinical information improved performance metrics of plasma p-tau181 and NfL, but not plasma Aβ_42_/Aβ_40_, classifiers by 15–23% (Table 3; column (B)). Plasma p-tau181 enhanced with clinical information perform similarly to plasma Aβ_42_/Aβ_40_. When further enhanced with the MRI-score in addition to the clinical information, classifier performance metrics for both plasma p-tau181 and plasma NfL increased by an additional 3–8%, with plasma p-tau181 performing slightly better with an accuracy of 82%, PPV of 83% and NPV of 82% (Table 3; column (C)). Similarly, the MRI-score enhanced classification performance of plasma Aβ_42_/Aβ_40_ more than clinical information (DeLong *P* < 10^−4^), reaching an AUC of 0.94 with an accuracy of 86%, PPV of 86% and NPV of 88%. The full classifier model, including all three plasma biomarkers, MRI-score, and clinical information achieved an AUC of 0.92 and an accuracy of 86%, with a PPV of 88% and NPV of 86%. This was not significantly different than the classifier model with plasma Aβ_42_/Aβ_40_, MRI-score and clinical information (DeLong *P* = 0.31), suggesting minimal added value of plasma NfL and plasma p-tau181. The most significant variables in a decreasing order of importance based on mean decrease in accuracy analysis were plasma Aβ_42_/Aβ_40_, MRI-score, *APOE* ε4 allele, age and CDR-SB.

**Table 3:** Performance of classifier models in differentiating Aβ+ individuals with mild CI

	(A) Plasma biomarkers						(B) Clinical information with and without plasma biomarkers						(C) MRI-score with and without clinical information and plasma biomarkers					
	AUC^a^	Acc	PPV	NPV	Sens	Spec	AUC^a^	Acc	PPV	NPV	Sens	Spec	AUC^a^	Acc	PPV	NPV	Sens	Spec
MRI score													0.76 [0.70, 0.82]	0.67 ± 0.02	0.70 ± 0.02	0.63 ± 0.03	0.72 ± 0.03	0.61 ± 0.04
Demographics + Clinical ^b^							0.81 [0.75, 0.87]	0.74 ± 0.02	0.76 ± 0.02	0.73 ± 0.03	0.81 ± 0.03	0.66 ± 0.05	0.88 [0.83, 0.92]	0.81 ± 0.02	0.82 ± 0.03	0.80 ± 0.03	0.85 ± 0.03	0.76 ± 0.04
Aβ42/Aβ40	0.87 [0.75, 0.99]	0.77 ± 0.06	0.79 ± 0.07	0.76 ± 0.08	0.79 ± 0.08	0.75 ± 0.10	0.85 [0.71, 0.99]	0.79 ± 0.05	0.81 ± 0.10	0.77 ± 0.06	0.80 ± 0.07	0.77 ± 0.14	0.94 [0.87, 1.00]	0.86 ± 0.05	0.86 ± 0.07	0.88 ± 0.08	0.90 ± 0.07	0.82 ± 0.1
p-tau181	0.64 [0.56, 0.71]	0.58 ± 0.03	0.63 ± 0.02	0.52 ± 0.03	0.61 ± 0.05	0.55 ± 0.05	0.85 [0.80, 0.90]	0.79 ± 0.02	0.80 ± 0.02	0.78 ± 0.04	0.83 ± 0.04	0.73 ± 0.03	0.90 [0.86, 0.94]	0.82 ± 0.02	0.83 ± 0.01	0.82 ± 0.04	0.86 ± 0.03	0.78 ± 0.02
NfL	0.56^c^ [0.49, 0.64]	0.54 ± 0.03	0.60 ± 0.02	0.48 ± 0.03	0.57 ± 0.03	0.51 ± 0.04	0.81 [0.75, 0.86]	0.73 ± 0.02	0.75 ± 0.03	0.71± 0.03	0.79 ± 0.04	0.66 ± 0.05	0.87 [0.83, 0.92]	0.81 ± 0.02	0.82 ± 0.03	0.79 ± 0.02	0.85 ± 0.03	0.75 ± 0.06
p-tau181 + NfL	0.70 [0.63, 0.77]	0.66 ± 0.02	0.69 ± 0.02	0.62 ± 0.02	0.72 ± 0.03	0.58 ± 0.05	0.84 [0.79, 0.89]	0.77 ± 0.03	0.78 ± 0.03	0.76 ± 0.04	0.83 ± 0.04	0.70 ± 0.06	0.89 [0.85, 0.93]	0.82 ± 0.02	0.83 ± 0.03	0.81 ± 0.03	0.86 ± 0.03	0.77 ± 0.05
Aβ42/Aβ40 + p-tau181 + NfL	0.88 [0.76, 0.99]	0.80 ± 0.05	0.81 ± 0.07	0.81 ± 0.08	0.84 ± 0.09	0.76 ± 0.11	0.89 [0.78, 1.00]	0.82 ± 0.06	0.85 ± 0.10	0.81 ± 0.07	0.83 ± 0.07	0.82 ± 0.13	0.92 [0.82, 1.00]	0.86 ± 0.05	0.88 ± 0.07	0.86 ± 0.06	0.87 ± 0.05	0.86 ± 0.09

To assess the added value of each class of variables (i.e. clinical, plasma and MRI classes), additional RF classifiers were constructed from (i) each plasma marker alone, (ii) each plasma marker jointly with clinical features, (iii) MRI-score jointly with clinical features and (iv) each plasma marker jointly with clinical features and MRI-score.

a 95% Confidence intervals.

b Demographics: age, sex, years of education and APOE ε4 status; Clinical assessments: MMSE, ADAS-Cog and CDR-SB.

c The confidence interval includes the axis *y* = *x*, suggesting that the classifier was not better than chance.

## Discussion

The major findings of this multi-centre biomarker study were (i) of the three plasma biomarkers, when considered separately, Aβ_42_/Aβ_40_ consistently differentiated PET Aβ-positivity status both in CU and in CI participants, with a slightly better performance in CIs, whereas plasma p-tau181 showed moderate value for differentiating PET Aβ-positivity status in CI participants, and plasma NfL lacked Aβ-positivity stratification value both in CU and in CI participants; (ii) clinical information, dominated by *APOE* ε4 status and education in CU participants, and by *APOE* ε4 status and age in CI participants, as well as MRI-score independently differentiated PET Aβ− and Aβ+ both in CU and in CI participants; (iii) clinical information enhanced the performance of plasma biomarkers in differentiating PET Aβ− and Aβ+ participants by 0.06–0.14 units of AUC for CUs, and by 0.21–0.25 units for CIs and (iv) further enhancement of these models with an MRI-score yielded an additional improvement of 0.04–0.11 units of AUC for CUs and 0.05–0.09 units for CIs. Taken together, the results recapitulate plasma Aβ_42_/Aβ_40_ as a robust biomarker of brain Aβ-positivity and suggest that when combined with clinical information, plasma p-tau181 and NfL biomarkers, and an MRI-score, could effectively identify Aβ+ individuals with expected greater accuracy in the symptomatic individuals. Interestingly, when the MRI-score is considered in combination with clinical information, plasma p-tau181 and plasma NfL have minimal added value for brain Aβ-positivity stratification in this multi-centre ADNI cohort of CU and CI participants.

### Plasma Aβ_42_/Aβ_40_ detects PET Aβ-positivity

The first major finding was that plasma Aβ_42_/Aβ_40_ was a robust biomarker of PET Aβ-positivity independent of clinical diagnosis, whereas plasma p-tau181 detected PET Aβ-positivity only in CIs with a moderate accuracy, and plasma NfL lacked value for stratification of PET Aβ+ and PET Aβ− cases both in CU and in CI cohorts. It should be noted that this finding was replicated when the modelling and testing of all classifiers were repeated on the plasma Aβ_42_/Aβ_40_ sub-cohort to mitigate the potential influence of sample size and sub-cohort characteristics in comparisons of classifiers (Supplementary Fig. 2). Of the three plasma biomarkers considered in this study, Aβ_42_/Aβ_40_ has been the most extensively studied in the literature. Recent studies, particularly the ones using highly sensitive mass spectrometry, have repeatedly reported a strong correlation between plasma Aβ_42_/Aβ_40_ and the gold-standard CSF and PET Aβ measures (Janelidze *et al.*, 2016; Ovod *et al.*, 2017; Nakamura *et al.*, 2018; Schindler *et al.*, 2019). Consistent with our findings, plasma Aβ_42_/Aβ_40_, especially when combined with age and *APOE* ε4 status, have been shown to accurately stratify Aβ+ individuals (e.g. AUC, 0.80–0.85) in the AD continuum (Palmqvist *et al.*, 2019b; Schindler *et al.*, 2019). The slightly superior performance of plasma Aβ_42_/Aβ_40_ in this study (cf. Supplementary Fig. 3) compared to the previous reports of 0.79–0.82 AUC for the detection of Aβ-positivity in CU participants (Fandos *et al.*, 2017; de Rojas *et al.*, 2018; Chatterjee *et al.*, 2019) and 0.90 AUC for CIs (Lin *et al.*, 2019) might be due to high molecular specificity and detection sensitivity of LC-MS/MS technique used to analyse the ADNI plasma samples. This observation is consistent with the notion that the different assays for plasma Aβ_42_/Aβ_40_ may have different precision and, in particular, mass spectrometry-based assays compared to immunoassays might be more accurate and robust in measuring levels of plasma Aβ species as biomarker of brain Aβ (Zetterberg, 2019). Another factor contributing to the high performance of the Aβ_42_/Aβ_40_ ratio, as compared with single biomarkers, is that between-individual differences in basal ‘total’ Aβ secretion are compensated for in the ratio, by dividing with Aβ_40_, whereas such differences in plasma NfL and p-tau181 levels, MRI measures or cognitive abilities might introduce variability in these measures.

### Plasma p-tau181 presented a more complex picture as a candidate biomarker of brain Aβ-positivity

Assays for the quantification of plasma p-tau181 are very recently developed (Zetterberg and Blennow, 2020) and are still under extensive investigation to fully understand the role of different plasma tau species as peripheral markers of AD pathophysiology. Compared to the limited number of previously reported evaluations of plasma p-tau181 as a biomarker of brain Aβ-positivity in other research and clinical cohorts (Mielke *et al.*, 2018; Palmqvist *et al.*, 2019b; Barthélemy *et al.*, 2020; Janelidze *et al.*, 2020a; Karikari *et al.*, 2020; Thijssen *et al.*, 2020), ADNI plasma p-tau181 levels measured by the Simoa assay differentiated between PET Aβ+ and PET Aβ− ADNI CI participants with an inferior accuracy (AUC, 0.64). Furthermore, this biomarker had no stratification value for PET Aβ-positivity within the ADNI CU participants (AUC, 0.55). The addition of clinical information to this base model increased AUC for the classification of Aβ+ versus Aβ− by 0.14–0.69 in CUs and by 0.21–0.85 in CIs. The subsequent addition of an MRI-score to this model further increased AUC for the classification of Aβ+ versus Aβ− by 0.11–0.80 in CUs and by 0.05–0.90 in CIs, bringing its classification performance to a clinically acceptable level.

Potential sources of the discrepancy between our results and those of other groups may include differences in the plasma analysis assays, diagnostic composition and demographic characteristics of the study cohorts, methodology used to determine ground-truth brain Aβ-positivity status and data analytics. One of the earliest plasma p-tau181 studies on a Meso Scale Discovery (MSD) platform reported that plasma p-tau181 as a good biomarker of the elevated brain Aβ with an AUC of 0.7 in CU and 0.85 in MCI participants in their discovery cohort but this study lacked internal validation or replication in an external validation cohort (Mielke *et al.*, 2018). Another study (Barthélemy *et al.*, 2020) reported high specificity of plasma p-tau181, measured by a highly sensitive mass spectrometry assay, for Aβ plaque pathology in their discovery cohort (*n *=* *34; including clinically diagnosed CU, MCI and AD individuals) and then replicated their findings with an AUC of 0.72 to differentiate Aβ− and Aβ+ individuals in an independent replication cohort of CUs, MCIs and ADs (*n *=* *92) but the performance within CU only or MCI only sub-cohorts was not statistically significant. Similarly, a larger multi-cohort study which included individuals with various clinical diagnoses including CU, MCI and AD reported a stepwise increase in plasma p-tau181 levels, measured on the MSD platform, with both Aβ-positivity and cognitive impairment and achieved an AUC of 0.81 in differentiating Aβ− and Aβ+ individuals, which was increased to 0.84 with the addition of plasma Aβ_42_/Aβ_40_ (Janelidze *et al.*, 2020a).

The age of cohort participants may also influence the ability of plasma p-tau181 to detect Aβ-positivity status. For instance, a multi-cohort study used the Simoa assay to measure plasma p-tau181 in four different cohorts (Karikari *et al.*, 2020) and found that plasma p-tau181 biomarker discriminated Aβ+ CU older adults and individuals with CI from Aβ− CU older adults and young adults with an AUC of 0.76–0.88 across cohorts. However, the CU older adults in this study were on average 10 years younger than ADNI participants, raising the question about age-dependent sensitivity of plasma p-tau181 to AD-related Aβ pathology. Similarly, another small cohort study of CU and CI participants, who were on average 13 years younger than ADNI participants, reported an excellent AUC of 0.86 in CU and 0.94, although not internally validated or replicated in an external cohort, in differentiating PET Aβ+ and PET Aβ− CIs with plasma p-tau181 levels (Thijssen *et al.*, 2020). It is highly likely that younger Aβ+ participants might have greater pathophysiological changes than the older ADNI participants in response to Aβ toxicity, which might be a driving factor for increased plasma p-tau181 levels. Indeed, it is well established that younger individuals who are Aβ+ have more brain tau deposition than older individuals who are Aβ+ (Schöll *et al.*, 2017). Furthermore, previous studies found that the strong correlations between plasma p-tau181 and Aβ PET are often in the Aβ+ but not in Aβ− individuals (Janelidze *et al.*, 2020a) and that increased plasma p-tau181 levels might be initiated by accumulation of Aβ beyond the positivity threshold, and continue to increase as Aβ further accumulates in the brain even during early stages of tau pathology as measured by Braak & Braak staging at autopsy or tau PET during life (Janelidze *et al.*, 2020a; Karikari *et al.*, 2020). Evidence from these recent studies together with the stronger association of plasma p-tau181 with brain Aβ burden in younger cohorts might suggest that plasma p-tau181 is unlikely to be a direct measure of Aβ pathology but instead a marker of tau pathology. Our finding that plasma p-tau181 has moderate stratification accuracy for PET Aβ-positivity only at the symptomatic disease stage suggests that p-tau181 detects Aβ-positivity only once a significant tau pathology, which is closely associated with symptoms, is detectable.

### Plasma NfL was a poor biomarker of PET Aβ-positivity

The relatively poor performance of plasma NfL in differentiating Aβ+ and Aβ− ADNI individuals, either symptomatic or asymptomatic, is largely consistent with the previous literature. Previous studies found no evidence that plasma NfL was related to Aβ or tau pathology as measured by PET or even synaptic dysfunction as measured by fluorodeoxyglucose-PET imaging, repeatedly emphasizing that plasma NfL is more likely to be a marker of all cause neurodegeneration (Mattsson *et al.*, 2019; Mielke *et al.*, 2019; Janelidze *et al.*, 2020a; Thijssen *et al.*, 2020). Finally, our finding that plasma p-tau181 and plasma NfL did not improve Aβ-positivity stratification accuracy above and beyond the plasma Aβ_42_/Aβ_40_ was consistent with the previous studies on other AD research cohorts (Palmqvist *et al.*, 2019b).

### Clinical information and MRI-score independently differentiated PET Aβ+ and Aβ− ADNI individuals

To date, the most common candidate predictors considered for Aβ-positivity were age, *APOE* genotype and measures of cognition, largely because they are easier to collect with widely available standardized protocols. Of these, age has been the most common predictor of elevated brain Aβ followed by the *APOE* genotype (reviewed in Ashford *et al.* (2021)), consistent with the notion that after advanced age, *APOE* ε4 genotype is a major risk factor for developing AD (Payami *et al.*, 1997). Consistent with the prior knowledge, age and *APOE* genotype were important predictors of Aβ-positivity for ADNI CU and CI participants (cf. Supplementary Fig. 3). In the main analyses, we observed that the ability of clinical information to differentiate Aβ+ and Aβ− participants improved, especially in terms of sensitivity, with increasing severity of clinical diagnosis. Indeed, measures of global cognition, such as MMSE and CDR-SB, had greater influence in the classifier model for Aβ-positivity within the CI participants. Consistent with our findings, accumulating evidence suggests that elevated Aβ is associated with risk of cognitive worsening and may indicate a pre-symptomatic stage of disease (Roe *et al.*, 2013; Donohue *et al.*, 2017). As the relationships between cognition and AD biomarkers are expected to be subtle, the global measures of cognition may have insufficient sensitivity among CUs to reliable detect pre-symptomatic expression of Aβ pathology, as reflected in our results with extremely low sensitivity of clinical information in detecting Aβ-positivity in CUs.

MRI-score of brain Aβ alone stratified Aβ+ and Aβ− participants with an AUC of 0.74 in ADNI CUs and an AUC of 0.76 in ADNI CIs with a substantially increased sensitivity. When combined with clinical information, MRI-score performed as well as, or, in CIs, even better than, the best performing plasma biomarker, Aβ_42_/Aβ_40_. Although structural T1-weighted MRI is not a molecular imaging modality directly targeting quantification of protein accumulation in the brain, MRI has been a gold standard for neurodegeneration (Jack *et al.*, 2004). The evidence for a relationship between Aβ deposition and neurodegeneration has been previously demonstrated in very early AD and even in asymptomatic individuals (Bourgeat *et al.*, 2010; Chételat *et al.*, 2010). In a similar manner to plasma p-tau181, the value of the MRI-score for Aβ-positivity might be a reflection of neurodegenerative processes due to Aβ toxicity, yet we observed that the MRI-score outperformed the plasma p-tau181. The brain Aβ deposition has a spatially distinct signature of cortical atrophy (Bourgeat *et al.*, 2010; Chételat *et al.*, 2010; Tosun *et al.*, 2011) and MRI-based correlates of brain Aβ deposition compared to plasma analytes might have the advantage of capturing this spatial information. Furthermore, although structural T1-weighted imaging has been traditionally considered to reveal fat and water distribution and distinguish tissue types, cellular changes associated with neuropathology might also influence the MRI contrast as well as the MRI intensity quality, such as the grey value distribution, texture features and spatial heterogeneity (Sørensen *et al.*, 2016; Feng *et al.*, 2019; Ranjbar *et al.*, 2019). Our results also suggest that deep learning, the computational approach used in this study to construct MRI-scores, might efficiently quantify Aβ toxicity from structural MRI because of its high automatic feature learning and visual pattern recognition abilities (LeCun *et al.*, 2015).

### Both clinical information and MRI-score enhanced performance of plasma biomarkers in identifying PET Aβ-positivity

One interesting observation was that although when combined with clinical information and MRI-score, plasma p-tau181 and NfL biomarkers could effectively identify Aβ+ symptomatic individuals, plasma p-tau181 and plasma NfL did not contribute to the detection of brain Aβ above and beyond the classification power of clinical information and MRI-score jointly, particularly in CUs. This is a particularly important criterion in the selection of candidate cost-effective and rapid screening tools for broad implementation in clinical and drug trial settings. Demographics and global cognitive measures are an integral part of the clinical assessment. MRI has long played a role in inclusion and exclusion criteria in patient recruitment and ruling out other causes of cognitive symptoms (Frisoni *et al.*, 2010). Furthermore, MRI has been routinely acquired in clinical trials to identify and monitor adverse events (Cash *et al.*, 2014). Plasma biomarkers, therefore, should have a classification ability as good as or better than clinical information and MRI separately and in combination in order to be a practical non-invasive screener.

Our results in this ADNI study, although limited to CU and CI participants, suggest that plasma Aβ_42_/Aβ_40_ but not plasma p-tau181 and plasma NfL might have added value in screening for brain Aβ-positivity. It is also important to emphasize that plasma assays target brain-derived proteins that are present at extremely low concentrations in the peripheral circulation and originate not only in the brain but almost all peripheral cells (Roher *et al.*, 2009). What plasma Aβ measures mean biologically and to what extent the variances seen in plasma Aβ levels reflect brain pathology especially in the CU and CI clinical groups in which greater heterogeneity in comorbid conditions is expected are questions still warrant further investigations. These limitations may make the use of the plasma Aβ biomarkers to predict the AD pathology more difficult at the individual level. Despite the inferior performance of plasma p-tau181 in detecting AD Aβ-positivity observed in this ADNI cohort, this biomarker may have different utility. Plasma p-tau181 can be robustly measured in plasma and is highly specific for AD pathology (Mielke *et al.*, 2018), making it an attractive screening tool for brain Aβ and tau pathologies jointly as required for A/T/N biomarker profiling (Jack *et al.*, 2018) linked to differential trajectories of disease progression (Altomare *et al.*, 2019; Jack *et al.*, 2019; Ebenau *et al.*, 2020). Further studies are warranted to better understand the behaviour of plasma p-tau181 as a biomarker of the burden of the disease at different disease stages (Lantero Rodriguez *et al.*, 2020). Given that Aβ-positivity assessment using either CSF or PET is independent of clinical diagnosis, clinical stage-dependent classifier performance might be a concern if these plasma biomarkers are operationalized in clinical practice. In our analysis, a similar clinical diagnosis-dependent gradual increase in classification performance was observed in Aβ-positivity classifier constructed with clinical information and to a lesser extent with MRI-score.

## Study design-specific considerations

There are multiple strengths to the study including the large sample size, well-characterized participants, and availability of plasma analytes, Aβ PET imaging and structural MRI, all assessed within a short period of time. A limitation of this *in vivo* study was the use of Aβ PET as the gold standard for brain Aβ-positivity rather than the true gold standard of neuropathology. A limitation of plasma analyte comparisons is that different techniques were used, namely Simoa for p-tau181 and NfL and LC-MS/MS for Aβ_42_/Aβ_40_. Despite the superior specificity, mass spectrometry has the disadvantage of being more expensive and requiring more expertise than immunoassays, which are easily analysed by laboratories that routinely run blood tests. Another limitation of the study is the potential pre-analytical variability since the blood samples were collected at multiple ADNI sites. Although the collection site as a categorical variable had no significant effect on ADNI plasma levels, multi-centre studies of plasma analytes still require further investigation for standardization of protocols to reduce measurement variability (Rozga *et al.*, 2019). We should also note that this study was limited to plasma p-tau181. Other blood immunoassays targeting tau species, specifically the very recently reported plasma pTau-217, might be promising biomarkers for AD Aβ pathology (Janelidze *et al.*, 2020b). Finally, we should further emphasize that this study is based on a convenience cohort where the degree of true population representation is not known. Most notable, about 47% of CU and 19% of CI ADNI participants who were CSF p-tau positive were PET Aβ−, suggesting non-AD aetiology of their tau pathology that might have particularly impacted the observed plasma p-tau181 levels (Benussi *et al.*, 2020). Additionally, the PPV and NPV performance of the classifier models considered in this study was limited by the prevalence of the PET Aβ-positivity in the selected ADNI cohort and may not be directly comparable to other studies with different PET Aβ-positivity prevalence.

## Conclusion

In summary, *in vivo* gold standard for brain Aβ burden assessment is currently Aβ PET or lumbar puncture for CSF Aβ_42_ (Tapiola *et al.*, 2009; Palmqvist *et al.*, 2016). The widespread acceptance of biomarker classification scheme for the AD continuum (Jack *et al.*, 2018) has ignited interest in more affordable and accessible approaches to detect AD Aβ pathology, a process that often slows down the recruitment into, and adds to the cost of, clinical trials. To this end, our systematic comparison of Aβ-positivity stratification models that use minimally invasive and low-cost measures identified demographics, *APOE* genotype, global cognitive measures, MR imaging, plasma Aβ measures, plasma p-tau181 and plasma NfL biomarkers, some alone and some in combination, as promising Aβ-positivity classifiers. Advances in ultrasensitive assays for plasma analytes as well as in computational classifier techniques combining multidisciplinary information further promise reduce the difficulty and cost of screening participants with AD Aβ pathology.

## Supplementary Material

fcab008_Supplementary_Data

## Notes

Data used in preparation of this article was obtained from the Alzheimer’s Disease Neuroimaging Initiative (ADNI) database (adni.loni.usc.edu). As such, the investigators within the ADNI contributed to the design and implementation of ADNI and/or provided data but did not participate in analysis or writing of this report. A complete listing of ADNI investigators can be found at: http://adni.loni.usc.edu/wp-content/uploads/how_to_apply/ADNI_Acknowledgement_List.pdf

## Abbreviations

A: *β* = β−amyloid
AD =: Alzheimer’s disease
ADAS-Cog =: Alzheimer's Disease Assessment Scale—Cognitive subscale
ADNI =: Alzheimer’s Disease Neuroimaging Initiate
AUC =: area under curve
CDR-SB =: Clinical Dementia Rating—Sum of Boxes
CI =: cognitively impaired
CSF =: cerebrospinal fluid
CU =: cognitively unimpaired
LC-MS/MS =: liquid chromatography tandem mass spectrometry
LONI =: Laboratory of Neuro Imaging
MMSE =: Mini–Mental State Examination
MSD =: Meso Scale Discovery
NfL =: neurofilament light
NPV =: negative predictive value
p-tau181 =: phosphorylated-tau at threonine-181
PPV =: positive predictive value
RF =: random forest
Simoa =: Single molecule array
SUVR =: standardized uptake value ratio
