# Supplementary material for: Detection of β-amyloid positivity in Alzheimer’s Disease Neuroimaging Initiative participants with demographics, cognition, MRI and plasma biomarkers
Source: Brain Commun. 2021 Feb 2;3(2):fcab008. doi: 10.1093/braincomms/fcab008 (PMC8023542; doi:10.1093/braincomms/fcab008)
Supplement: fcab008_Supplementary_Data [file fcab008_supplementary_data.docx]

**Supplementary Materials**

**Aβ PET imaging**

The radiochemical synthesis of florbetapir for Aβ PET imaging was overseen and regulated by Avid Radiopharmaceuticals and distributed to the qualifying ADNI sites. PET imaging was performed at each ADNI site according to standardized protocols, as described online (http://adni.loni.usc.edu/methods/pet-analysis-method/pet-analysis/). All PET scans underwent a rigorous quality control protocol and were processed to produce final images with standard orientation and voxel size of 2 mm^3^ (Jagust *et al.*, 2015).

**Apolipoprotein E (*APOE*) genotyping**

For ADNI-1 DNA samples, *APOE* genotyping was carried out by polymerase chain reaction (PCR) amplification, Hhal restriction enzyme digestion, and subsequent standard gel resolution and visualization processes (Hixson and Vernier, 1990; Reymer *et al.*, 1995). For ADNI-GO and ADNI-2 DNA samples, genotyping was performed by Prevention Genetics (Marshfield, WI, USA) and LGC Genomics (Beverly, MA, USA), employing array processing using allele-specific PCR with universal molecular beacons and competitive allele-specific PCR, enabling bi-allelic scoring of single nucleotide polymorphisms (SNPs), respectively(Myakishev *et al.*, 2001; Hawkins *et al.*, 2002).

**Plasma sample collection**

The plasma samples were collected at the participating ADNI centers. After overnight fasting, plasma was collected in the morning by venipuncture into Vacutainer tubes (Becton Dickenson, Franklin Lakes, NJ) containing potassium K3 ethylene tetraacetate as an anticoagulant. After centrifugation, samples were placed in transfer tubes (13 mL polypropylene, Sarstedt Inc., Newton, NC, catalog number 60.541), frozen, and shipped on dry ice to the UPenn Biomarker Core Laboratory, where they were stored temporarily at −80°C. The average time from blood collection to freezing of plasma for shipment was 67 ± 41 minutes (95% confidence interval [CI]: 21–180 minutes). Within several weeks of receipt, the samples were thawed, aliquoted by 500 μL into aliquot tubes (1.5 mL polypropylene, Thermo Fisher Scientific, Waltham, MA, catalog number 05-408-129), and stored at −80°C pending biochemical analyses.

**ADNI Plasma Aβ_42_ and Aβ_40_ processing**

Due to issues like ‘clogging’ on LC/MS and contamination noise signal on MS detector, three ADNI specific processing steps were implemented and validated, as follows:

1. Ion trap filtering MS method, which allows quant lower amount of Aβ isoforms.
2. Decrease the plasma volume from 1.8mL to 0.45mL – reducing matrix effect, while maintaining signal strong enough for required accuracy.
3. Centrifugation prior to immunoprecipitation and using automated immunoprecipitation platform.

| 1. Cognitively unimpaired (CU) cohort | |
| --- | --- |
| 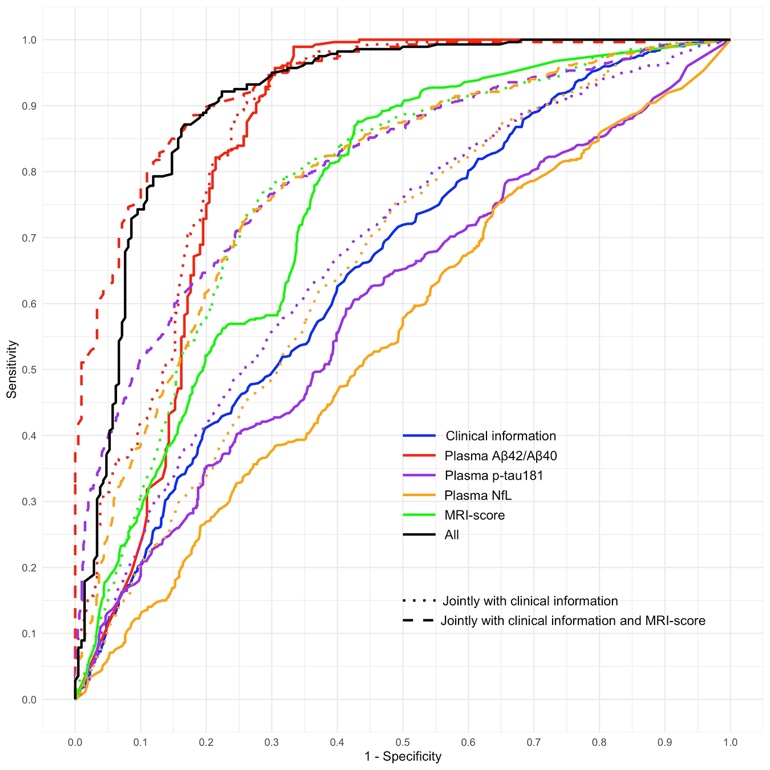 | 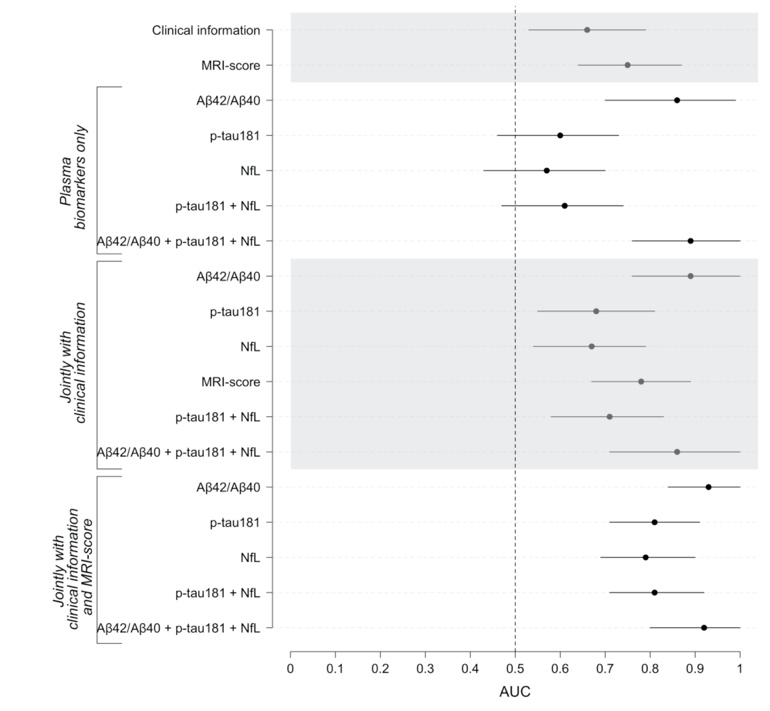 |
| 1. Mild cognitive impairment (CI) cohort | |
| 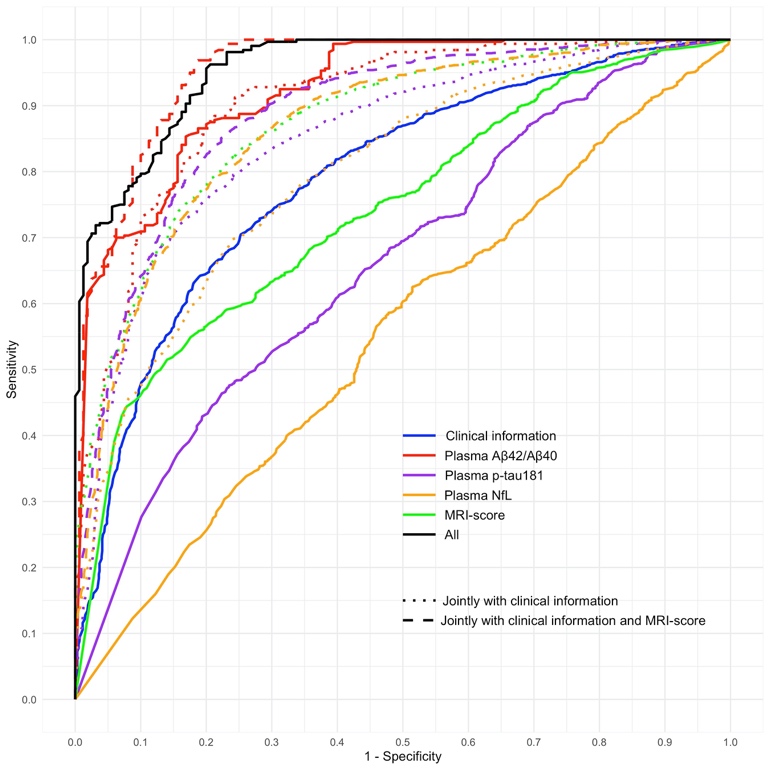 | 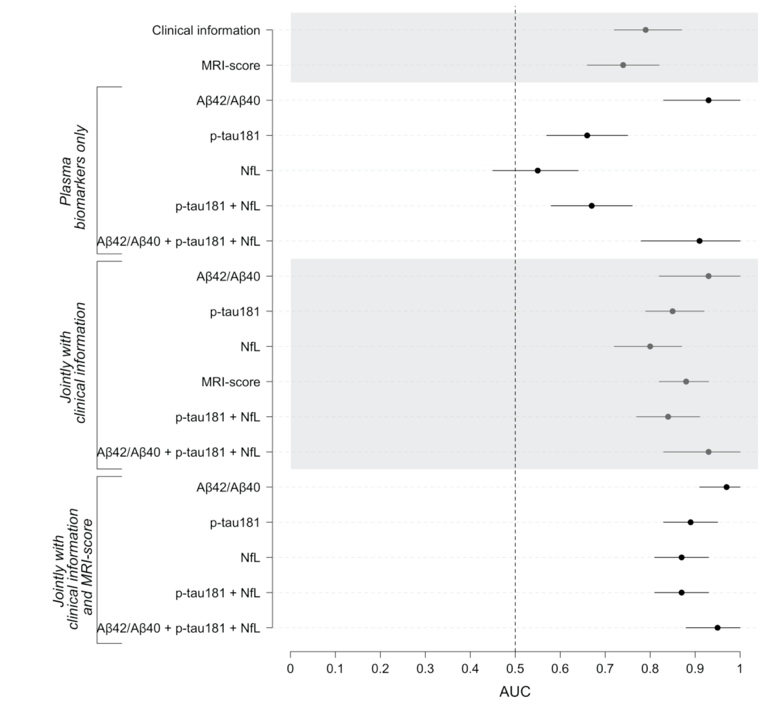 |
| **Supp Figure 1.** **Performance with CSF Aβ-positivity as the ground truth:** Receiver operating characteristic (ROC) analysis of Aβ positivity prediction in an ADNI cohort of a) cognitively unimpaired (CU) individuals and b) individuals with mild cognitively impairment (CI). Optimized ROC curves and corresponding areas under the curve (AUCs) for classifiers constructed separately and jointly with demographic information (age, sex, and years of education), *APOE*, clinical scores, plasma biomarkers (Aβ_42_/Aβ_40_, p-tau181, and NfL), and structural MRI–score when predicting Aβ-positivity using PET Aβ as the ground truth in the ADNI study. Error bars indicate union of 95% CIs from cross-validation iterations. | |

| 1. Cognitively unimpaired (CU) cohort | |
| --- | --- |
| 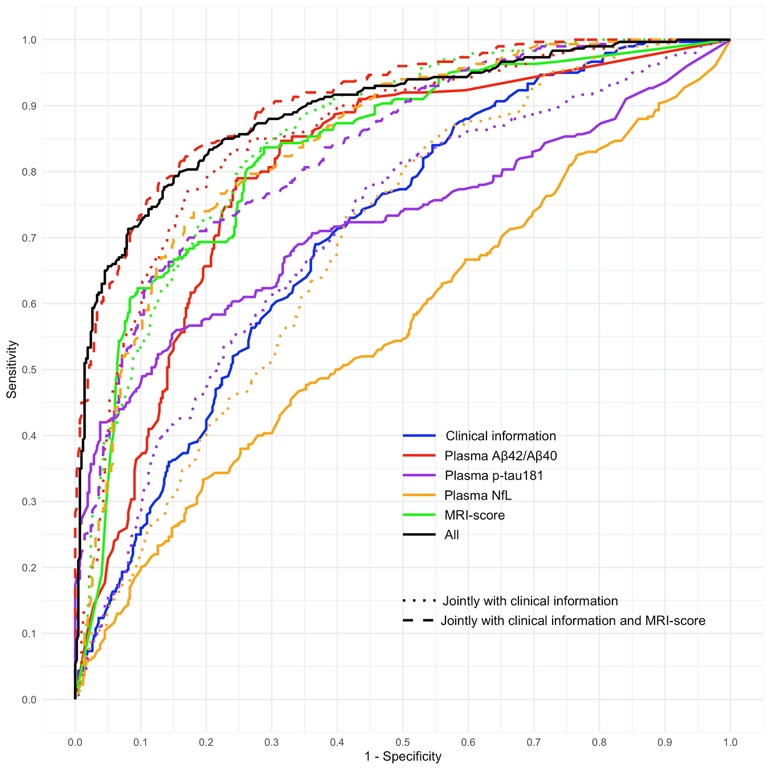 | 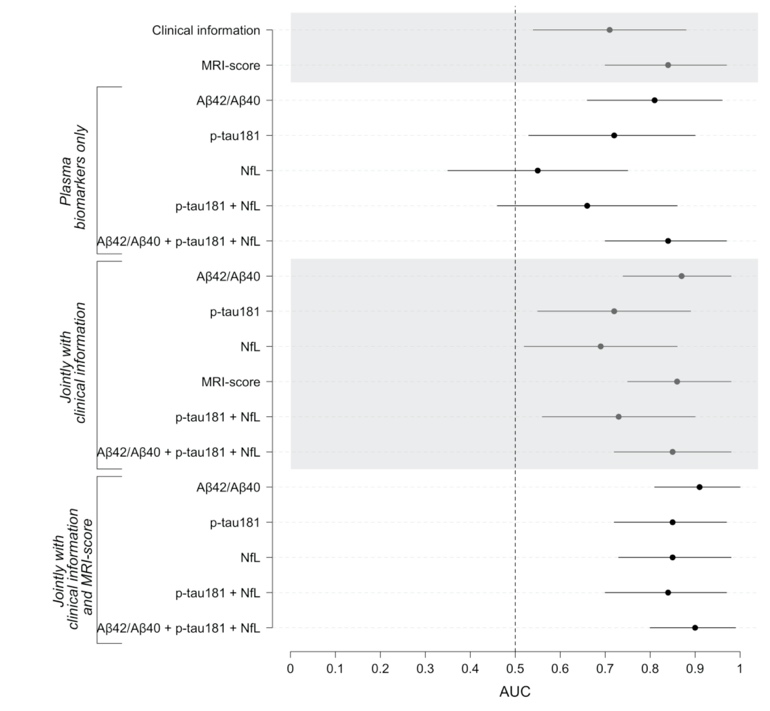 |
| 1. Mild cognitive impairment (CI) cohort | |
| 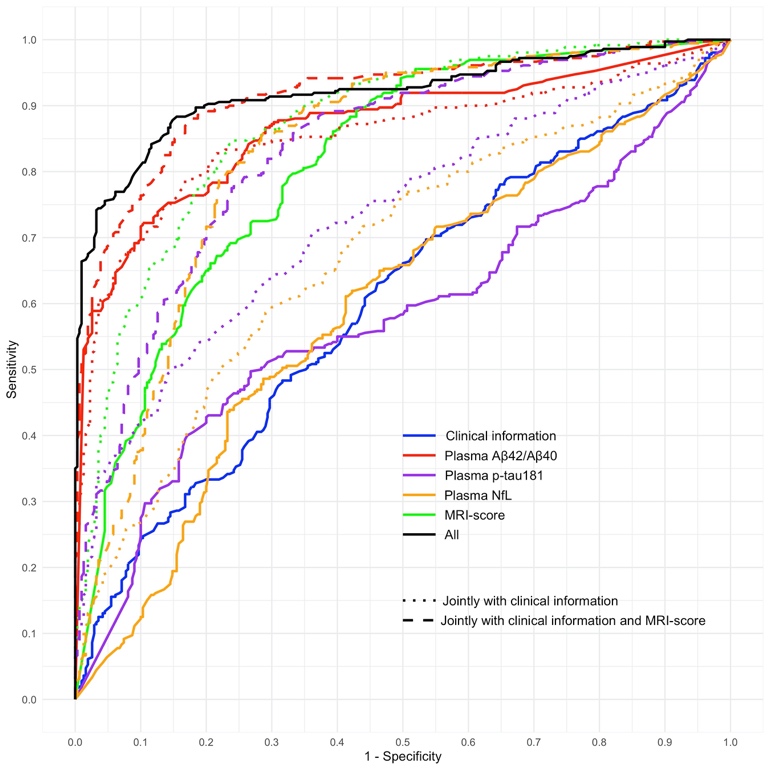 | 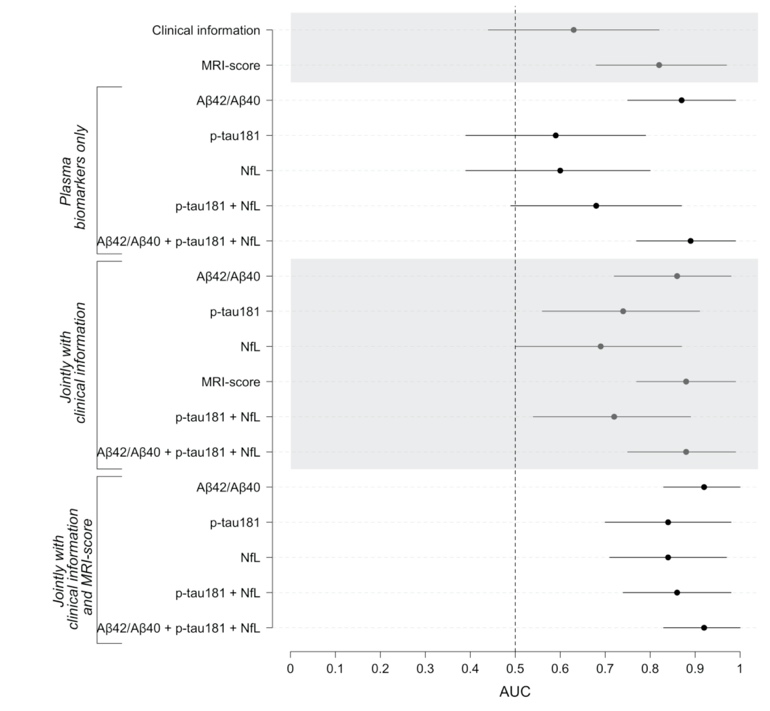 |
| **Supp Figure 2.** **Performance with plasma Aβ_42_/Aβ_40_ sub-cohort:** Receiver operating characteristic (ROC) analysis of Aβ positivity prediction in an ADNI cohort of a) cognitively unimpaired (CU) individuals and b) individuals with mild cognitively impairment (CI). Optimized ROC curves and corresponding areas under the curve (AUCs) for classifiers constructed separately and jointly with demographic information (age, sex, and years of education), APOE, clinical scores, plasma biomarkers (Aβ_42_/Aβ_40_, p-tau181, and NfL), and structural MRI–score when predicting Aβ-positivity using florbetapir PET as the ground truth in the ADNI study. Models were limited to sub-cohort of cases with plasma Aβ_42_/Aβ_40_ data, including n=87 CUs and n=86 CIs. Error bars indicate union of 95% CIs from cross-validation iterations. | |

| 1. Cognitively unimpaired (CU) cohort | |
| --- | --- |
| 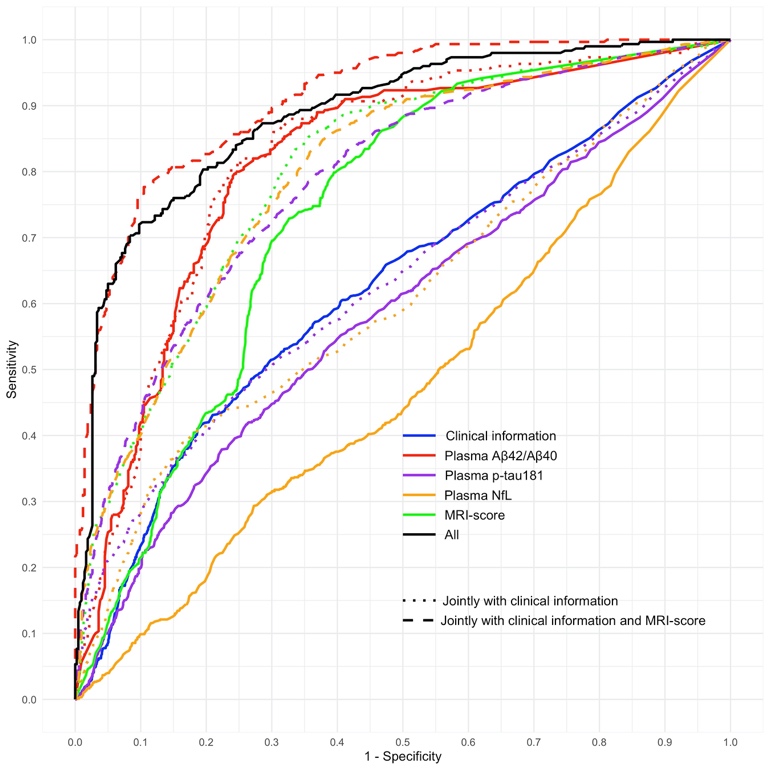 | 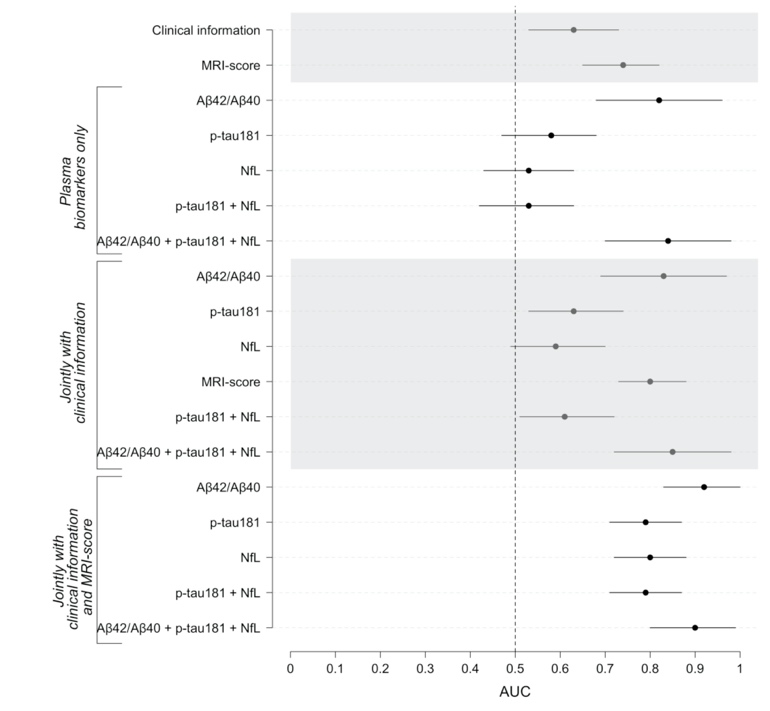 |
| 1. Mild cognitive impairment (CI) cohort | |
| 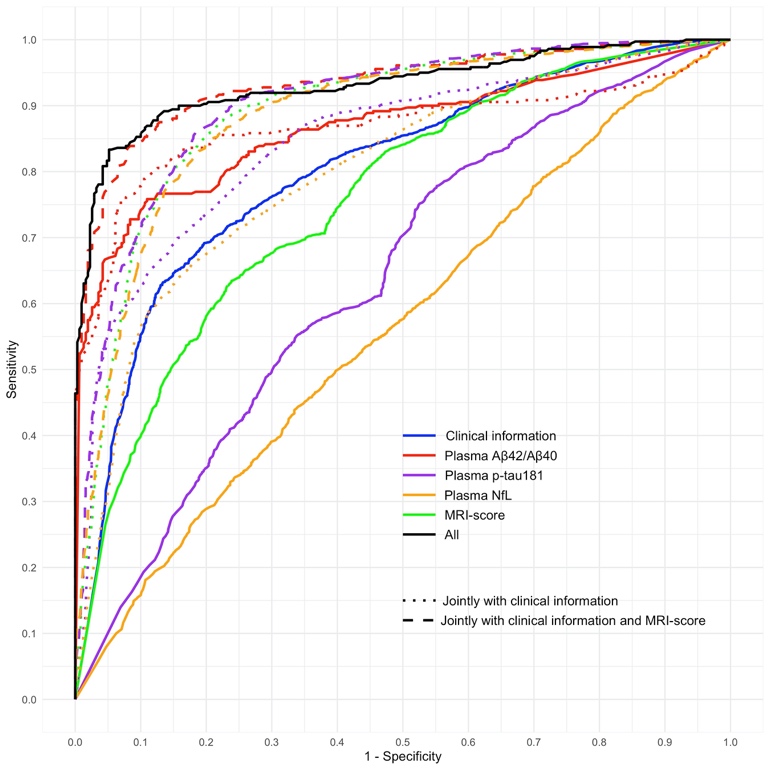 | 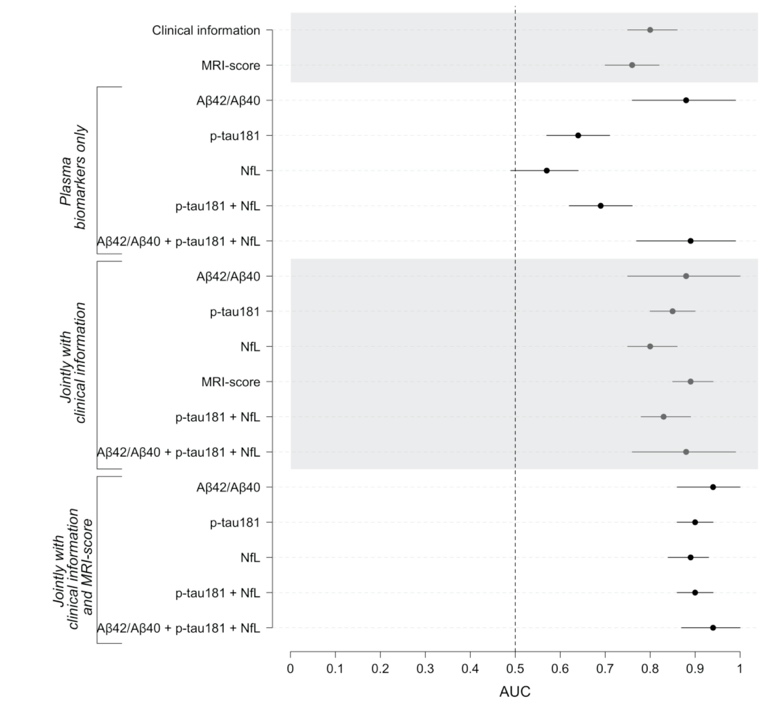 |
| **Supp Figure 3.** **Performance with clinical information limited to age and *APOE* genotype:** Receiver operating characteristic (ROC) analysis of Aβ positivity prediction in an ADNI cohort of a) cognitively unimpaired (CU) individuals and b) individuals with mild cognitively impairment (CI). Optimized ROC curves and corresponding areas under the curve (AUCs) for classifiers constructed separately and jointly with clinical information (age and *APOE* only), plasma biomarkers (Aβ_42_/Aβ_40_, p-tau181, and NfL), and structural MRI–score when predicting Aβ-positivity using PET Aβ as the ground truth in the ADNI study. Error bars indicate union of 95% CIs from cross-validation iterations. | |

| a) Cognitively unimpaired (CU) cohort | b) Cognitively impaired (CI) cohort |
| --- | --- |
| 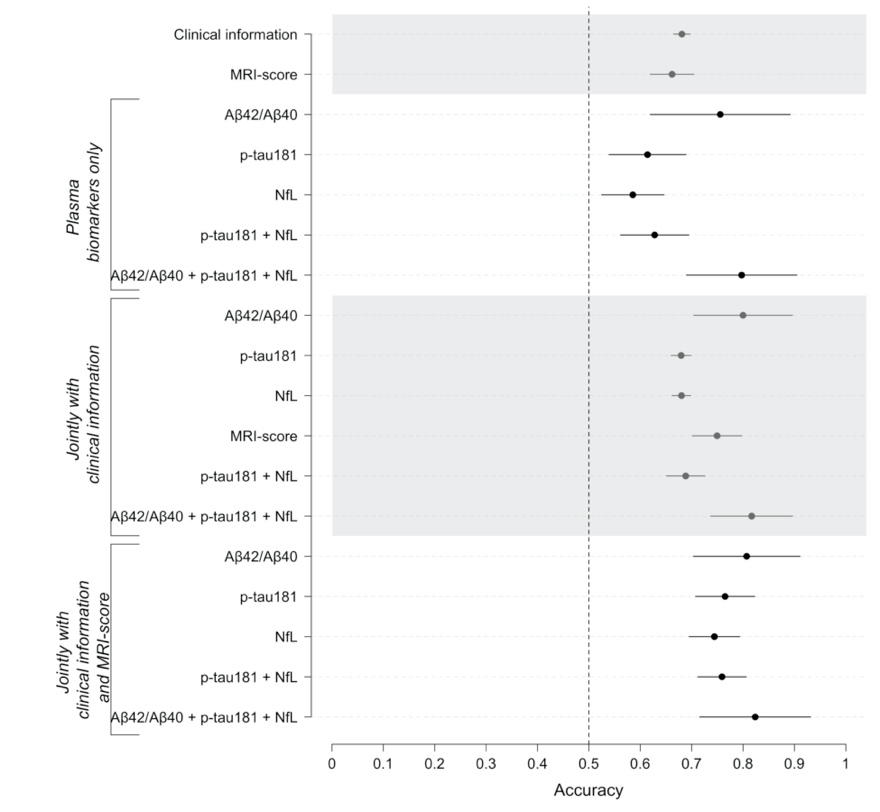 | 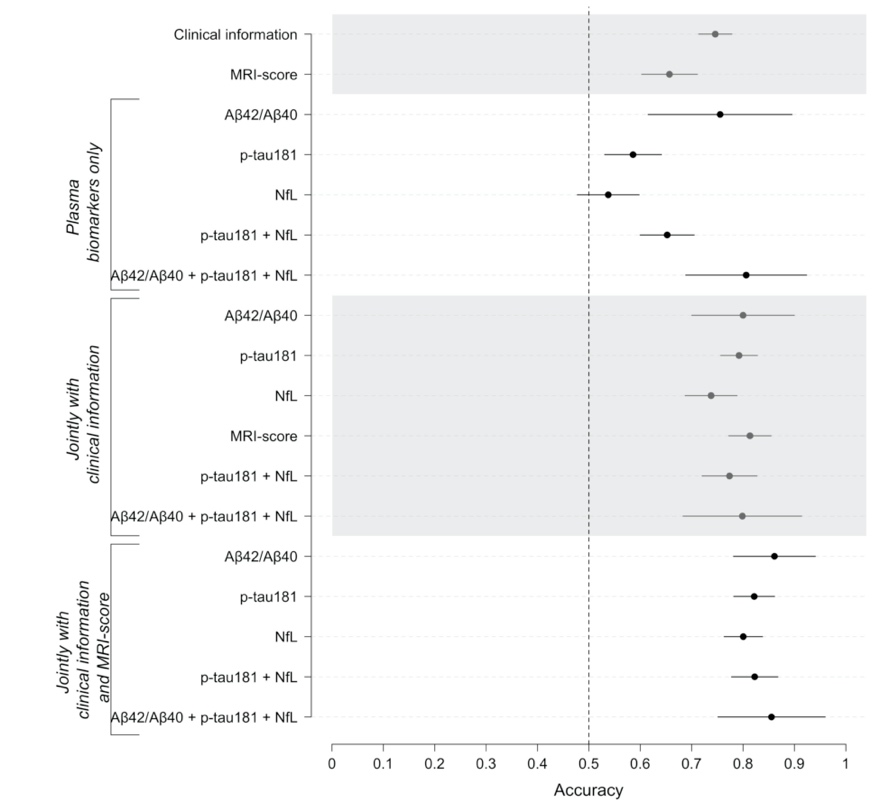 |
| 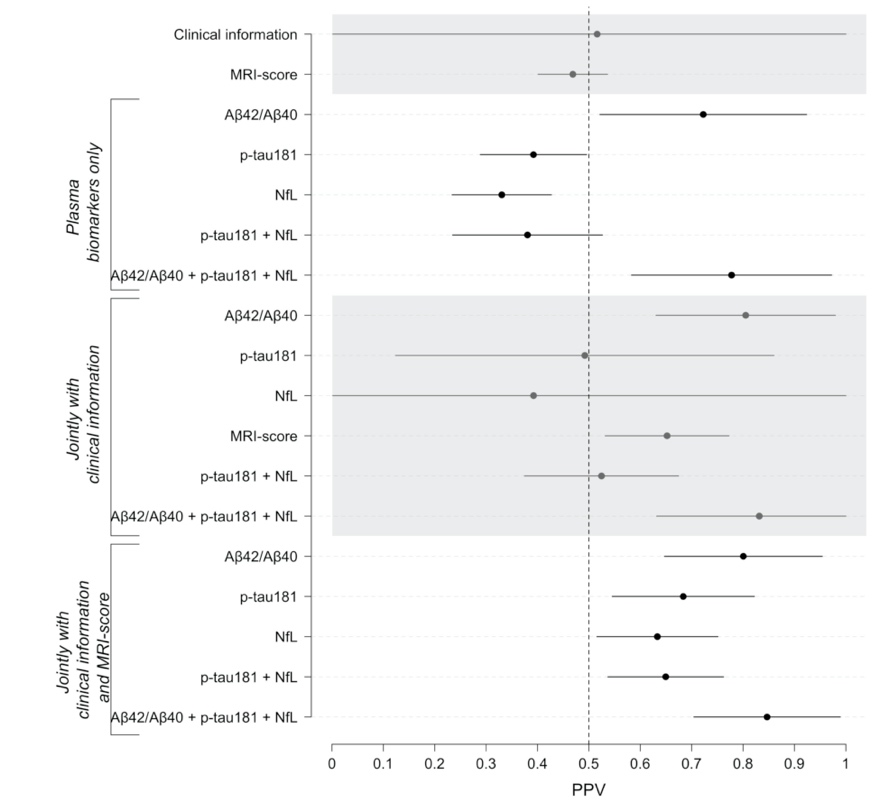 | 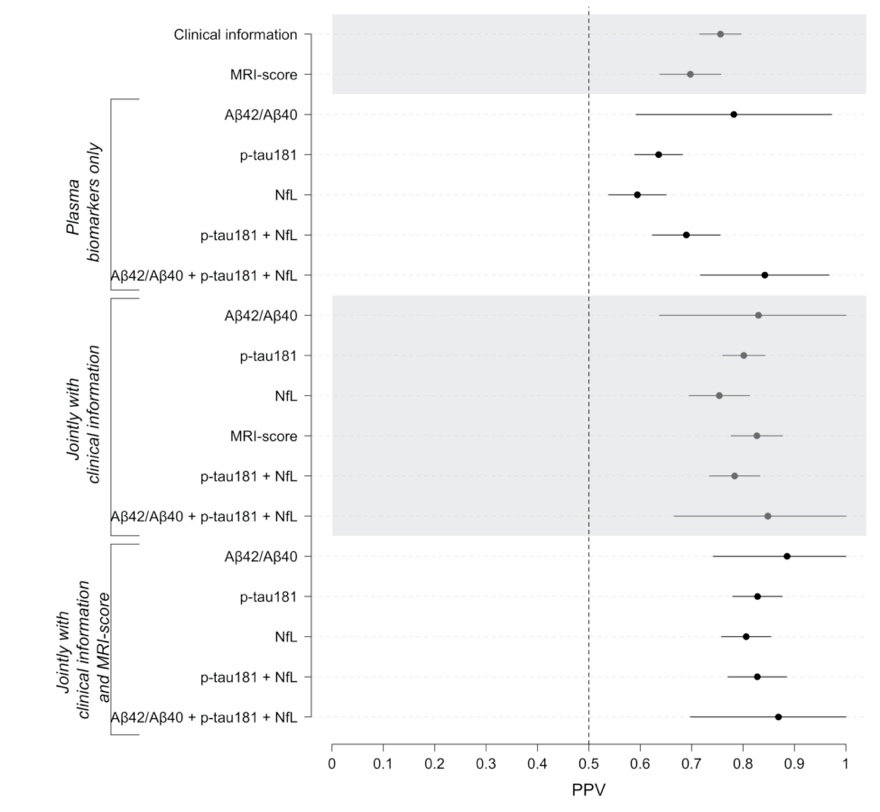 |
| 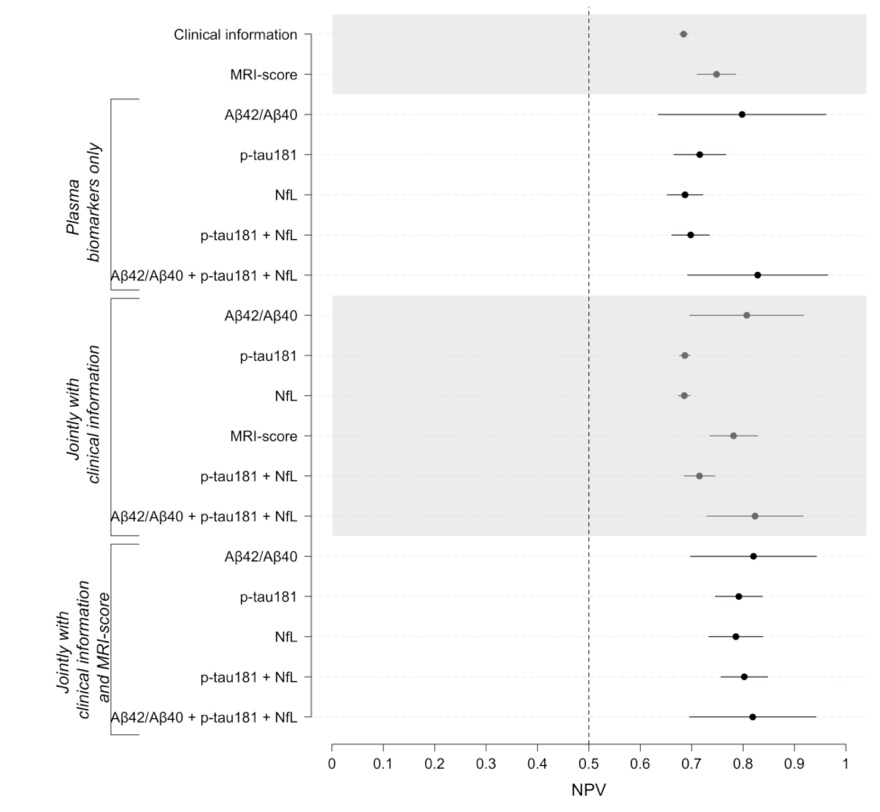 | 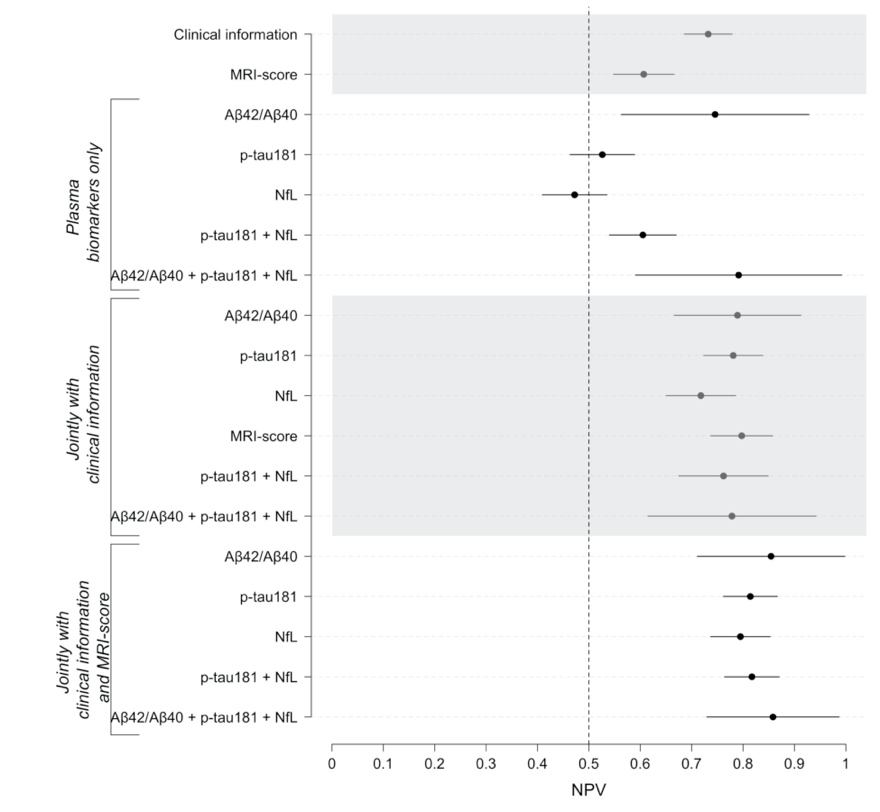 |
| 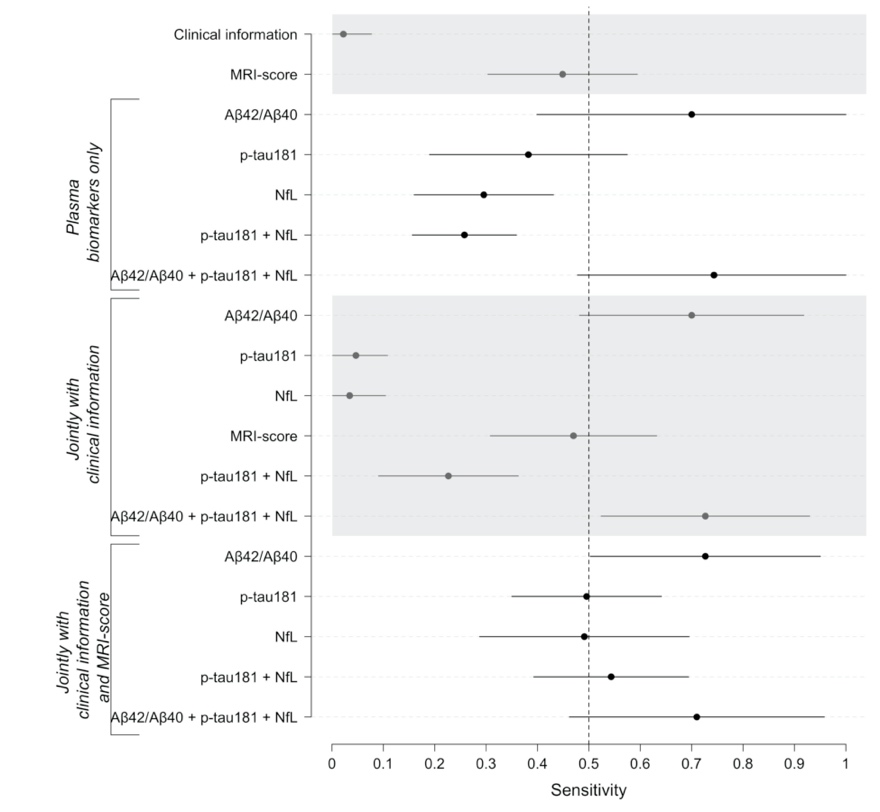 | 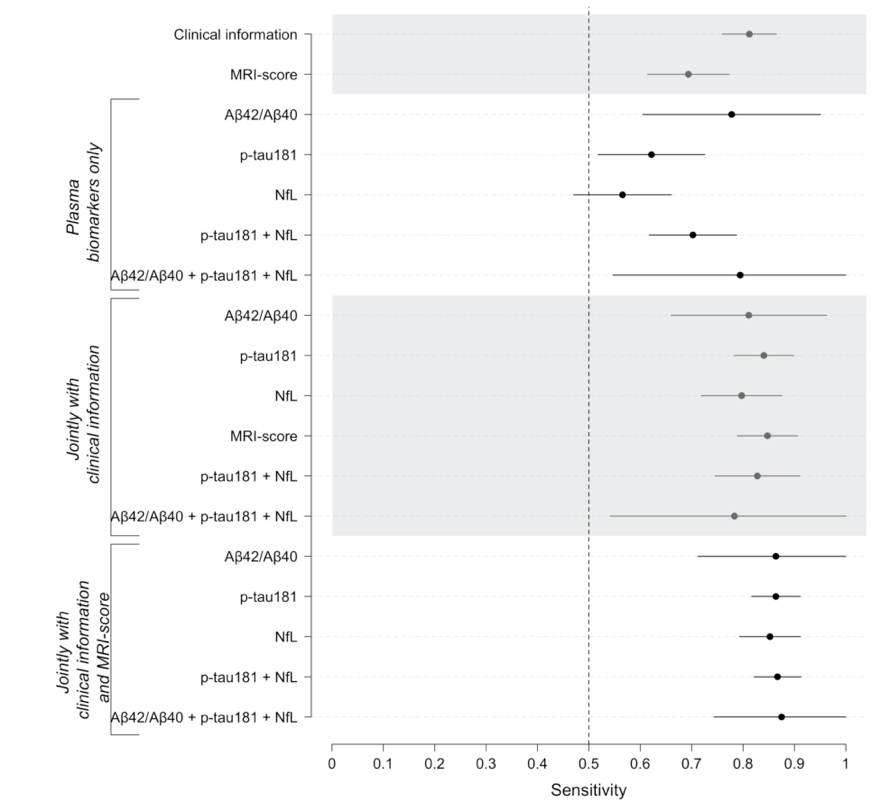 |
| 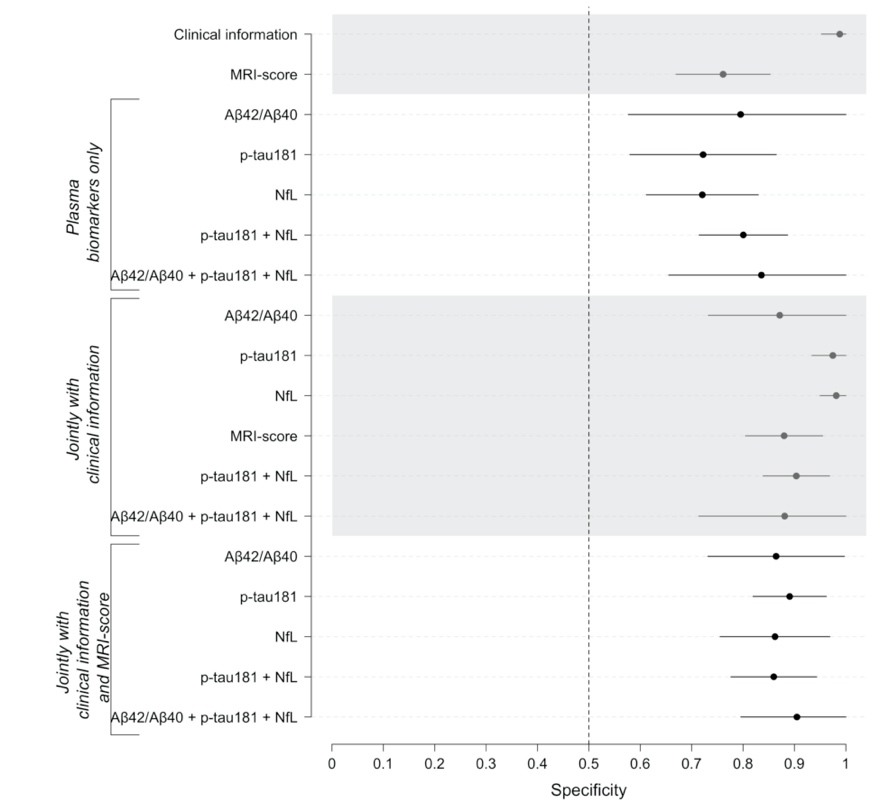 | 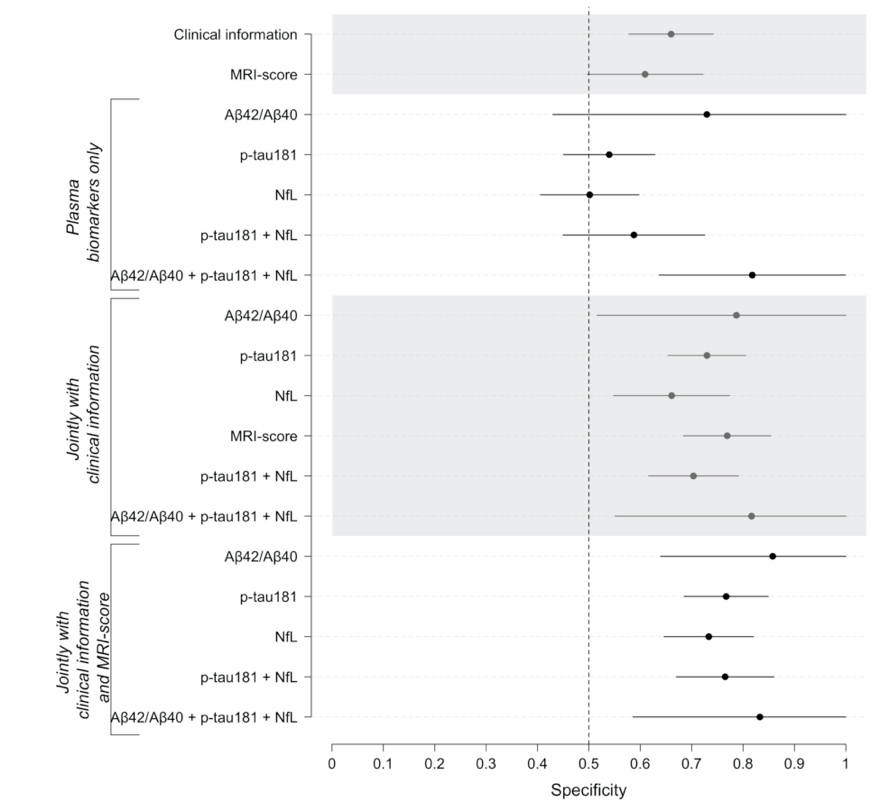 |
| **Supp Figure 4**. **Classifier performance metrics of Aβ positivity prediction** in A) cognitively unimpaired (CU) individuals and B) individuals with mild cognitively impairment (CI). Classification accuracy, positive predictive value (PPV), negative predictive value (NPV), sensitivity, and specificity estimates with ± 2 x standard variation error bars from cross-validation iterations are shown. | |

**References**

Hawkins JR, Khripin Y, Valdes AM, Weaver TA. Miniaturized sealed-tube allele-specific PCR. Hum Mutat 2002; 19(5): 543-53.

Hixson JE, Vernier DT. Restriction isotyping of human apolipoprotein E by gene amplification and cleavage with HhaI. J Lipid Res 1990; 31(3): 545-8.

Jagust WJ, Landau SM, Koeppe RA, Reiman EM, Chen K, Mathis CA*, et al.* The Alzheimer's Disease Neuroimaging Initiative 2 PET Core: 2015. Alzheimer's & dementia : the journal of the Alzheimer's Association 2015; 11(7): 757-71.

Myakishev MV, Khripin Y, Hu S, Hamer DH. High-throughput SNP genotyping by allele-specific PCR with universal energy-transfer-labeled primers. Genome Res 2001; 11(1): 163-9.

Reymer PW, Groenemeyer BE, van de Burg R, Kastelein JJ. Apolipoprotein E genotyping on agarose gels. Clin Chem 1995; 41(7): 1046-7.
